# Gradients of neurotransmitter receptor expression in the macaque cortex

**DOI:** 10.1038/s41593-023-01351-2

**Published:** 2023-06-19

**Authors:** Sean Froudist-Walsh, Ting Xu, Meiqi Niu, Lucija Rapan, Ling Zhao, Daniel S. Margulies, Karl Zilles, Xiao-Jing Wang, Nicola Palomero-Gallagher

**Affiliations:** 1 Computational Neuroscience Unit, Faculty of Engineering, University of Bristol, Bristol, UK; 2 Center for Neural Science, New York University, New York, NY USA; 3 Child Mind Institute, New York, NY USA; 4 Institute of Neuroscience and Medicine (INM-1), Research Centre Jülich, Jülich, Germany; 5 Integrative Neuroscience and Cognition Center, University of Paris Cité, Paris, France; 6 Cécile and Oskar Vogt Institute for Brain Research, Medical Faculty, University Hospital Düsseldorf, Heinrich Heine University Düsseldorf, Düsseldorf, Germany

**Keywords:** Computational neuroscience, Synaptic transmission, Genetics of the nervous system, Neurotransmitters

## Abstract

Dynamics and functions of neural circuits depend on interactions mediated by receptors. Therefore, a comprehensive map of receptor organization across cortical regions is needed. In this study, we used in vitro receptor autoradiography to measure the density of 14 neurotransmitter receptor types in 109 areas of macaque cortex. We integrated the receptor data with anatomical, genetic and functional connectivity data into a common cortical space. We uncovered a principal gradient of receptor expression per neuron. This aligns with the cortical hierarchy from sensory cortex to higher cognitive areas. A second gradient, driven by serotonin 5-HT_1A_ receptors, peaks in the anterior cingulate, default mode and salience networks. We found a similar pattern of 5-HT_1A_ expression in the human brain. Thus, the macaque may be a promising translational model of serotonergic processing and disorders. The receptor gradients may enable rapid, reliable information processing in sensory cortical areas and slow, flexible integration in higher cognitive areas.

## Abstract (web summary)

Froudist-Walsh et al. reveal organizational principles of receptor densities in macaque cortex. Densities of multiple receptor types align with changes in dendritic properties, myelin and functional networks. Data are openly released to the community.

## Main

A key challenge in neuroscience is to discover how a relatively static brain anatomy can adapt to a changing world. The brain’s connectivity is a key component. Mapping brain connectivity (the connectome) exhaustively across species is a major ongoing advance^1–5^. However, connectivity alone is insufficient to explain neural circuit dynamics underlying brain functions. The functional impact of synaptic connections depends on receptors. Thus, connectivity approaches, which are blind to receptor types, may not be sufficient to understand the computational capabilities of the cortex. To complement ongoing efforts to map the connectome, a systematic map of receptor densities across cortex is needed. This would provide a crucial link between the molecular and systems organization of the cortex.

The cortex displays a very similar regional and laminar receptor profile in macaques and humans^6^. Neuroanatomists have quantitatively mapped mesoscopic and microscopic anatomical details across the macaque cortex^3,7,8^. This currently outpaces our knowledge of human anatomy. In contrast, in vivo neuroimaging is less advanced in macaques than in humans. Nonetheless, recent developments in macaque neuroimaging can accelerate interspecies comparison and translation^9^. However, few studies have yet integrated in vitro neuroanatomy with in vivo neuroimaging^10–15^. In particular, receptor data are usually displayed in in vitro slices and seldom openly available. Creating openly accessible maps of receptor expression across the cortex that integrate with neuroimaging data could speed up translation across species and levels of neuroscience.

At the level of microcircuits, different regions of cortex share a common organization. However, their properties vary across the cortex in the form of macroscopic gradients^16^. Gradients of connectivity have been used to demonstrate the organizational structure of the cortex^17^. Similar understanding of the brain’s large-scale neurotransmitter and receptor organization is beginning to emerge. In the mouse brain, subcortical neuromodulatory centers are ‘connector hubs’^18^. Receptor expression in the human brain is associated with in vivo connectivity patterns and functional activity patterns^19–21^. However, it is not yet known how receptor expression relates to anatomical organization that we can measure in the macaque cortex.

In this study, we measured the density of 14 types of neurotransmitter receptors across 109 areas of macaque cortex. We mapped these data and multiple types of anatomical and functional data onto a common cortical space. These other data types included neuron density, dendritic tree size, spines, tract-tracing connectivity, gene expression and structural and functional magnetic resonance imaging (MRI). We found that the receptor architecture of macaque cortex can be well described by a small number of gradients. The principal receptor gradient defines a putative cortical hierarchy. Cortical areas high on the gradient had a higher density of receptors per neuron, larger dendrites and less myelin. Receptor gradients also segregated functional networks. This suggests a potential role for neuromodulators in propagating activity along cortical hierarchies and between higher cognitive networks.

## Results

### Distribution of receptors across macaque cortex

We analyzed receptor expression in the macaque brain using in vitro receptor autoradiography. This uses radioactive ligands to quantify the endogenous receptors in the cell membrane. Our analysis included 14 receptor types (glutamate: AMPA, kainate and NMDA; GABA: GABA_A_, GABA_A_/BZ and GABA_B_; acetylcholine: M_1_, M_2_ and M_3_; serotonin: 5-HT_1A_ and 5-HT_2A_; noradrenaline: α_1_ and α_2_; and dopamine: D_1_). We extracted receptor densities from regions defined by cyto and receptor architecture. Within each brain region, receptor densities were highly consistent across sections (median coefficient of variation = 0.08; Supplementary Fig. 1a) and across subjects (median coefficient of variation = 0.18; Supplementary Fig. 1b). Borders between brain regions are usually accompanied by a change in expression of two or more receptors. For example, changes in α_1_ and 5-HT_1A_ receptor density highlight the border between LIPd and LIPv (Supplementary Fig. 2; ref. ^22^). In the raw data, several receptors reached their highest densities in primary visual cortex (Supplementary Fig. 3a; GABA_A_, acetylcholine M_2_ and serotonin 5-HT_2A_; ref. ^23^). Many other receptors reached their highest densities in parts of the anterior cingulate. This includes all glutamatergic receptors, GABA_B_, serotonin 5-HT_1A_, noradrenaline α_1_ and dopamine D_1_ (Supplementary Fig. 3). M_1_, GABA_A_/BZ and α_2_ receptors are notable for having high densities in both cingulate cortex and V1. Thus, some common patterns of expression are seen across several receptors.

Serotonin 5-HT_1A_ has the steepest gradient of expression of all receptors. Generally, receptor density (per milligram of protein) varied by a factor of 1–5 across cortical areas. The receptor with the shallowest gradient in raw expression is serotonin 5-HT_2A_. The area with the greatest density of 5-HT_2A_ receptors has a density just 1.67 times higher than the area with the lowest density. In contrast, the peak expression of 5-HT_1A_ in area a24′ab of anterior cingulate cortex is over 17 times the density of 5-HT_1A_ receptors in area V1 (Supplementary Fig. 3b).

**Fig. 1:**
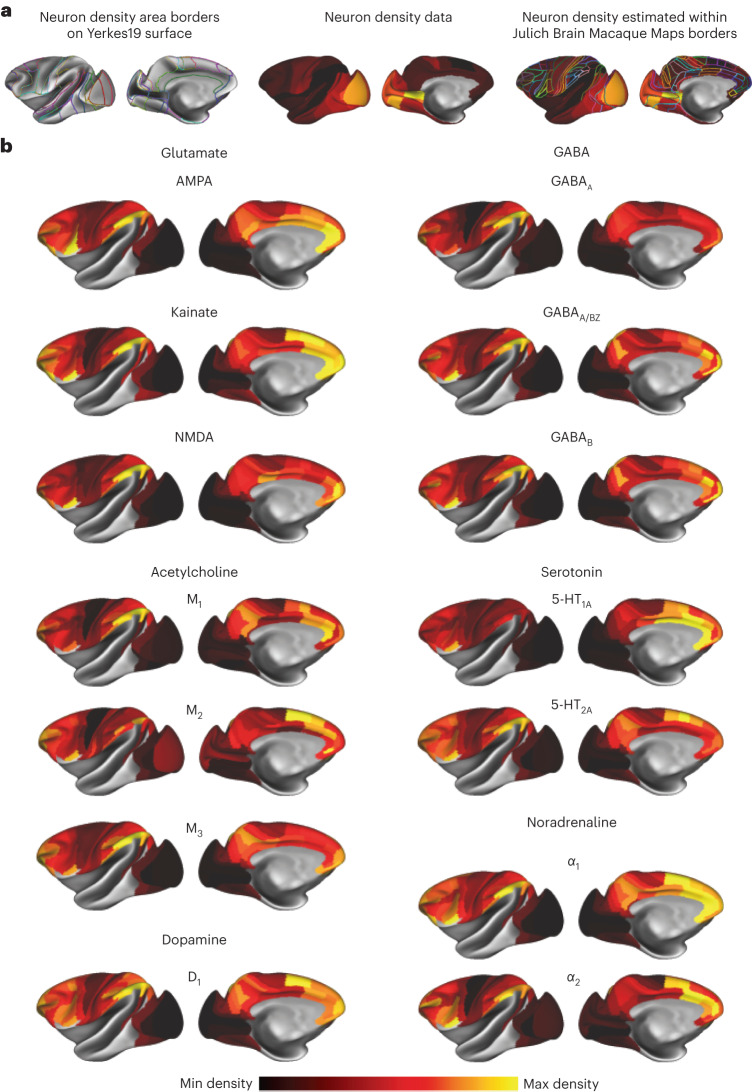
The density of 14 receptors per neuron across macaque cortex. **a**, Neuron density data from ref. ^8^ were delineated on the cortex and used to normalize receptor data. **b**, The receptor density per neuron of 14 receptor types assessed with in vitro receptor autoradiography.

The high density of receptors in V1 is due to its high neuron density. Neuron density varies by a factor of 5 across macaque cortex (Supplementary Fig. 3b). We mapped the receptor data and published neuron density data^8^ to the Yerkes19 cortical surface^24^. We estimated the receptor density per neuron across the cortex for all 14 receptor types (Fig. 1, Supplementary Tables 1 and 2 and Supplementary Fig. 4). Notably, although the density of several receptors peaks in V1 in the raw data, this is mostly erased when accounting for neuron density.

### The principal receptor gradient of macaque cortex

**Fig. 2:**
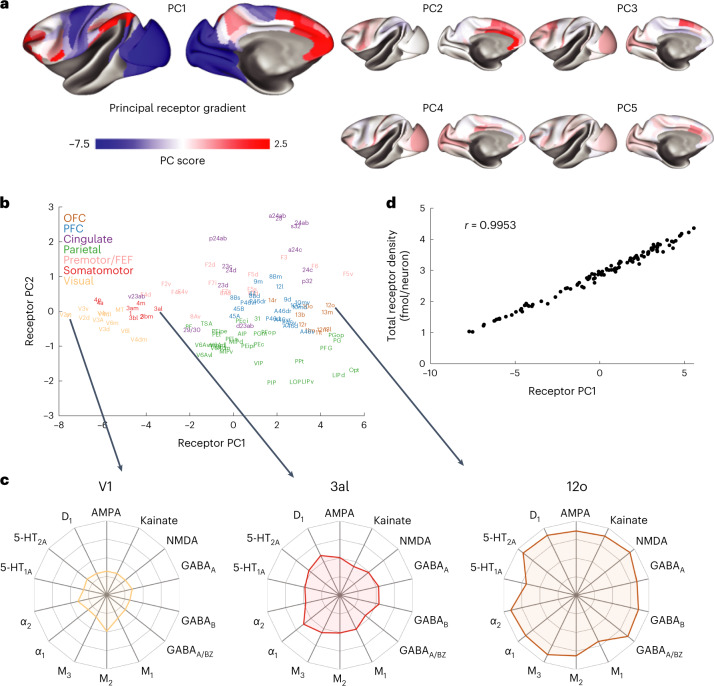
The principal receptor gradient captures total receptor density per neuron across macaque cortex. **a**, The first five PCs of the receptor per neuron data. **b**, The projection of brain regions onto the first two PCs of the receptor data (‘receptor space’). Brain regions are colored according to anatomo-functional groups for visualization purposes. **c**, The receptor fingerprints of three areas at different points along the first PC (that is, the principal receptor gradient). The density of most receptors increases along the gradient, from area V1 to 3al and again to 12o. For information on standard deviations, see Supplementary Table 2. **d**, The first PC closely follows the total receptor density per neuron. Pearson correlation, *r*(107) = 0.9953 (range: lower/upper bound of 95% confidence interval = 0.9931/0.9968, two-sided *P* = 4 × 10^−110^). OFC, orbitofrontal cortex; PFC, prefrontal cortex.

The principal receptor gradient spreads from early sensory to higher cognitive regions. Τo identify the main patterns of receptor expression, we performed a principal component analysis (PCA). We performed this on both the data per neuron (Fig. 2) and the raw data (Supplementary Fig. 5). The principal receptor gradient (PC1) explains 81% of the variance in the receptor data (per neuron). The top five principal components (PCs) explain over 95% of the variance (Fig. 2a and Supplementary Fig. 6; PCs 1–5 explain 81.2%, 6.5%, 3.5%, 2.4% and 1.4%, respectively).

All receptors contribute similarly to the principal receptor gradient (Supplementary Figs. 6c and 7). The gradient dependence is a measure of how much the spatial pattern of a gradient changes upon removal of one receptor type from the dataset. The principal receptor gradient is robust to removal of any individual receptor (Supplementary Fig. 7). Thus, the principal receptor gradient captures strong underlying trends across receptors. In contrast, serotonin 5-HT_1A_ expression drives the spatial pattern of PC2 (Supplementary Fig. 7). Similarly, acetylcholine M_2_ contributes strongly to PC3. M_2_ and dopamine D_1_ contribute to PC4 and noradrenaline α_2_, acetylcholine M_1_ and other receptors to PC5 (Supplementary Fig. 7).

Brain regions within the same functional system have similar receptor expression. We projected the data onto the first two PCs, which defined a ‘receptor space’. Visual, somatomotor, premotor, parietal, cingulate, prefrontal and orbitofrontal each occupy distinct sections of the receptor space (Fig. 2b). In Fig. 2b, we colored cortical areas according to these groupings for visualization purposes only.

The raw receptor gradients are similar to the receptor-per-neuron gradients. Each of the top five PCs of the raw receptor data (Supplementary Fig. 5a) is strongly correlated with the corresponding PC of the receptor-per-neuron data (Supplementary Fig. 5b). One difference is that V1 (which has a very high neuron density) shifts from the bottom of PC1 (per neuron data) to the bottom of PC2 (raw data). Below, we show that PC1 or PC2 in the receptor-per-neuron data correlates with various features of anatomy and function. Each of these correlations also holds for either PC1 or PC2 of the raw receptor data (Supplementary Fig. 8).

### Receptor expression increases along the principal gradient

Receptor fingerprints show the pattern of receptors expressed in each cortical area^25^. The size of the receptor fingerprint increases along the principal receptor gradient (Fig. 2c, areas V1, 3al and 12o). This shows an increase in receptor density per neuron across almost all receptors. The receptor-per-neuron density is four times higher in areas at the top of the gradient than in areas at the bottom (Fig. 2d). The principal receptor gradient closely tracks the total receptors per neuron across brain areas (Fig. 2d).

### The principal gradient aligns with the cortical hierarchy

**Fig. 3:**
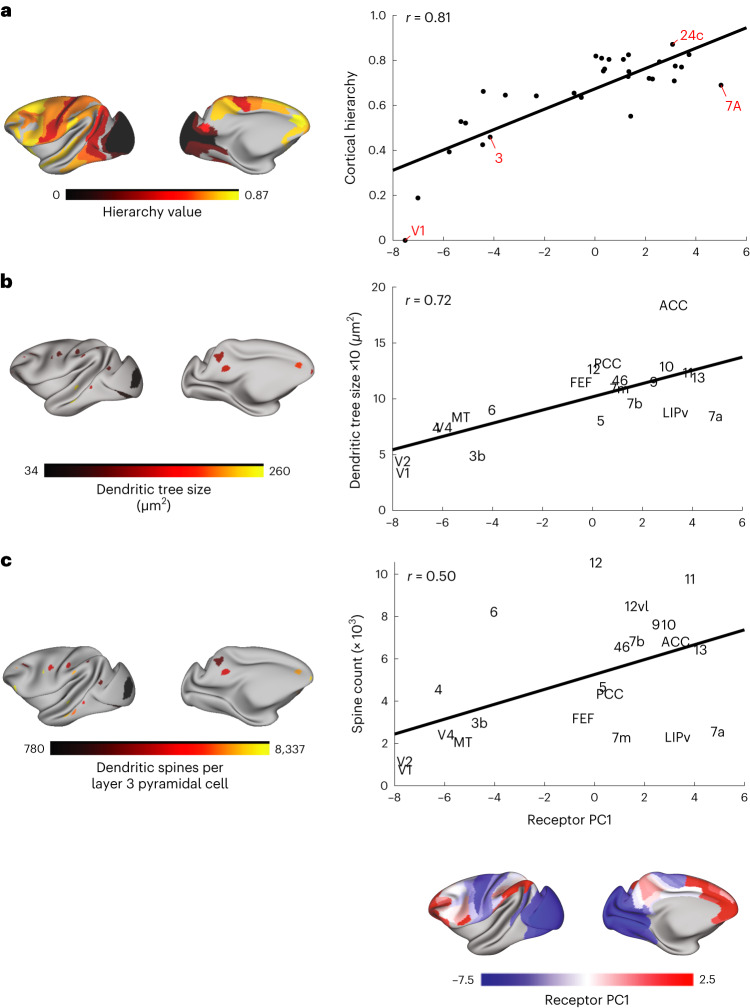
The anatomical foundations of the principal receptor gradient. **a**, There is a strong positive correlation between the principal receptor gradient and the cortical hierarchy (Pearson correlation, *r*(39) = 0.81 (range 0.64–0.90), *P* < 0.0001, corrected for spatial autocorrelation and multiple comparisons (Bonferroni)). **b**, Dendritic tree size is positively correlated with the principal receptor gradient (Pearson correlation *r*(19) = 0.73 (range: 0.43–0.88), *P* = 0.005, corrected for spatial autocorrelation and multiple comparisons (Bonferroni)). **c**, The relationship between the receptor principal receptor gradient and spine count is not significant after correction for spatial autocorrelation and multiple comparisons (Pearson correlation, *r*(20) = 0.54 (range: 0.15–0.78), *P* = 0.23, corrected for spatial autocorrelation and multiple comparisons (Bonferroni)).

The cortical hierarchy is anatomically defined by laminar connectivity patterns^26^. Functionally, the hierarchy spreads from areas that process simple sensory stimuli to areas that integrate varied and highly processed information. Could the receptor profile differ according to these distinct computational roles? Using retrograde tract-tracing data, we recently calculated the hierarchy of 40 cortical areas^27^. Here, we found that the principal receptor gradient is strongly correlated with the cortical hierarchy (Fig. 3a). Thus, neurons near the top of the hierarchy, which contribute to more complex functions, express more receptors.

### Dendritic trees increase in size along the principal gradient

How could neurons high in the principal receptor gradient house more receptors? Pyramidal cells receive the vast majority of their synaptic inputs on their dendrites. Thus, we hypothesized that dendritic properties would change along the principal receptor gradient. Elston et al.^7^ measured dendritic properties across dozens of areas of macaque cortex. We focused on the size of the dendritic tree and the number of dendritic spines on layer 3 pyramidal cells, for which most data were available. We mapped the locations for the dendritic injections to the Yerkes19 cortical surface (Fig. 3b,c). We found that the principal receptor gradient is positively correlated with dendritic tree size (Fig. 3b). The correlation between the principal receptor gradient and the total number of dendritic spines per neuron did not remain significant when accounting for the spatial autocorrelation and multiple comparisons (Fig. 3c). No significant correlation was observed between the principal receptor gradient and the density of spines per micrometer (*r*(20) = 0.28 (range: −0.16 to 0.63), *P* = 0.21, uncorrected). Thus, neurons at the top of the principal receptor gradient contain larger dendrites. This likely provides the neural real estate required to house a greater number of synaptic connections and receptors.

### An inverse relationship between myelin and receptor density

**Fig. 4:**
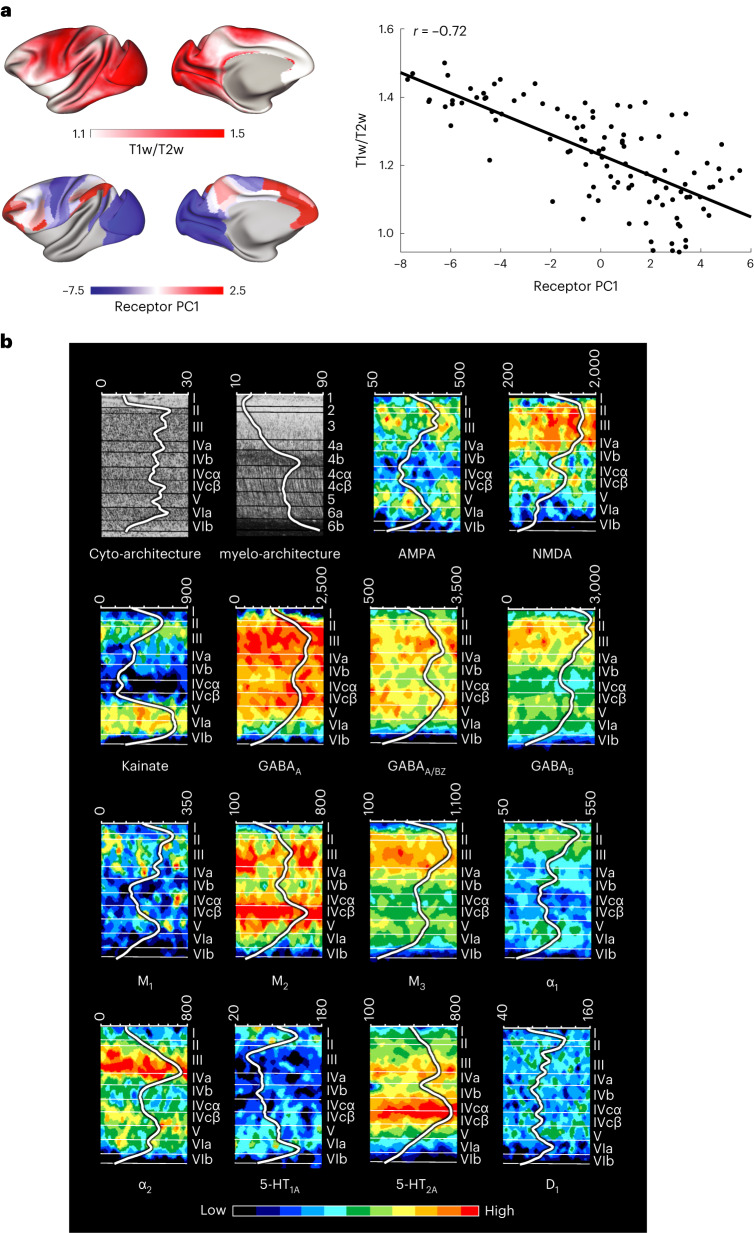
An inverse relationship between cortical myelin and receptor density. **a**, Cortical T1w/T2w ratio, a proposed marker for myelin content, is strongly negatively correlated with the principal receptor gradient across 109 cortical areas (*r*(107) = −0.72 (range: −0.80 to −0.62), *P* = 0.02, corrected for spatial autocorrelation and multiple comparisons (Bonferroni)). **b**, Receptor-, cyto-architecture and myelo-architecture of the macaque primary visual cortex (V1). Changes in Gray Level Index, which represents a measure of the volume fraction of cell bodies, in myelin density and in receptor concentration (in fmol mg^−^^1^ of protein) throughout the cortical depth are provided by the profile curve overlaid onto each section. Note that the scale has been optimized for each profile to provide the best visualization of changes in receptor densities throughout the cortical ribbon. Roman and Arabic numerals indicate cyto-architectonic and myelo-architectonic layers, respectively. Positions of cyto-architectonic layers were transferred to the neighboring receptor images.

Myelin inhibits synapse formation, axonal growth and experience-dependent plasticity^28^. The ratio of T1-weighted to T2-weighted (T1w/T2w) MRI signal is a proposed marker of myelination in the cortical gray matter. We analyzed T1w/T2w data from Donahue et al.^24^. We found that there is a strong negative correlation between the principal receptor gradient and the T1w/T2w ratio (Fig. 4a).

We also compared the densities of all 14 receptors across cortical layers to the pattern of laminar myelination using a histological myelin stain. We performed this comparison in primary visual area V1 (Fig. 4b), primary motor area 4a (Supplementary Fig. 9) and association parietal area PEipe (Supplementary Fig. 9). In areas 4a and PEipe, all receptors (except kainate and 5-HT_2A_ in PEipe) had higher densities in the supragranular layers (that is, I–III; Supplementary Fig. 9). In V1, most receptors had highest densities in layers II and III, followed by layer V and lowest densities in layers I, VI and IVb. Low receptor densities in layers I and VI may reflect the low cell densities in those layers (Fig. 4b). Some receptors, including NMDA, GABA_A_, GABA_B_, M_2_ and 5-HT_2_, also had high densities in layer IVc (Fig. 4b). In contrast, myelin is higher in the infragranular layers (that is, V–VI) than the supragranular layers. Additionally, V1 contains a high level of myelin in granular layer IVb. Thus, the receptor density pattern is opposite to the myelin density pattern across layers. Therefore, receptor expression may be constrained by myelination across cortical regions and layers.

### Principal gradient separates sensory and cognitive networks

**Fig. 5:**
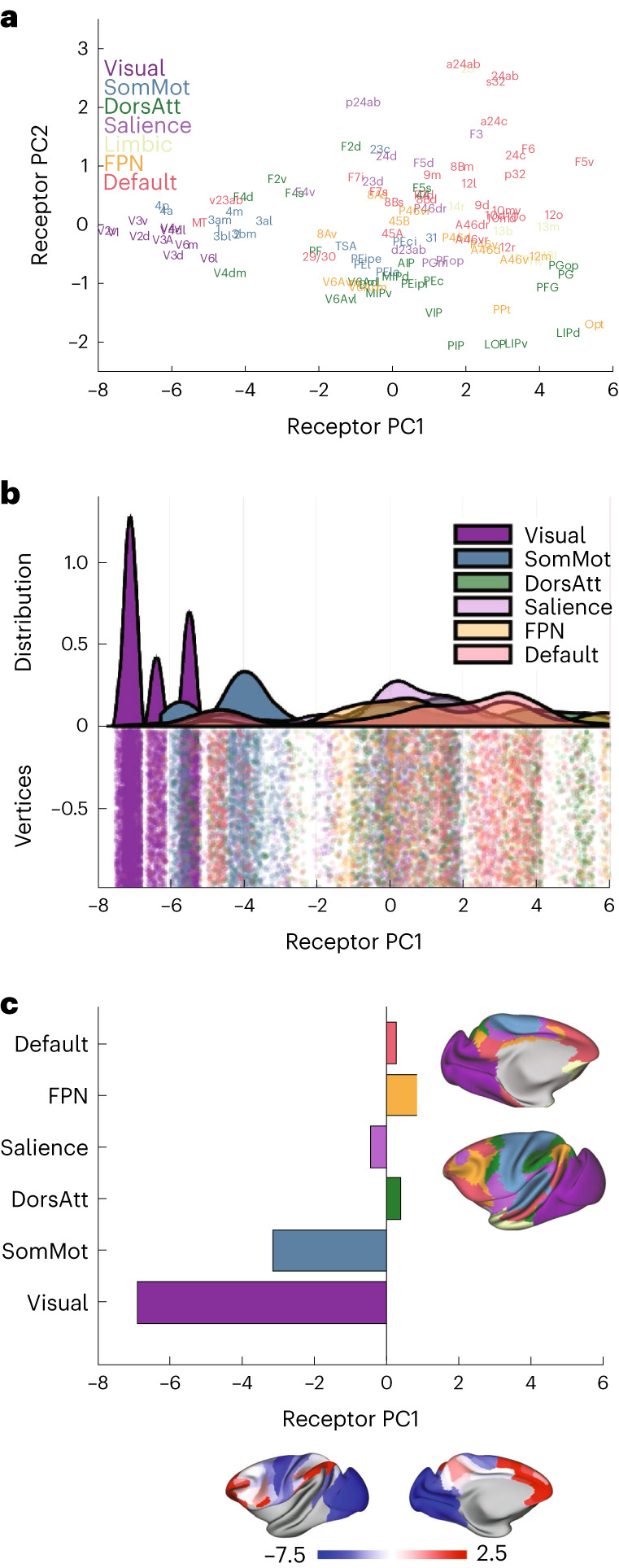
The principal receptor gradient separates sensory and cognitive networks. The canonical cognitive networks of Yeo et al.^30^ mapped to the macaque cortex by Xu et al.^29^ using cross-species functional alignment. **a**, The cognitive networks mapped into receptor space. **b**, The distribution of receptor PC1 scores for vertices in each cognitive network shown on a raincloud plot^75^. The ‘limbic’ network was excluded owing to a lack of receptor data. **c**, The mean PC score of the principal receptor gradient within each cognitive network. DorsAtt, dorsal attention network; FPN, frontoparietal network; SomMot, somatomotor network.

Does the principal receptor gradient shape in vivo interactions between cortical areas? Xu et al.^29^ used connectivity gradients to map from the human cortex to corresponding points on the macaque cortex. This method was used to align seven canonical cognitive networks^30^ to the macaque cortex (Fig. 5). We used this alignment to identify the receptor gradient expression across cognitive networks. The overlap of each area of the Julich Brain Macaque Maps with the seven cognitive networks is quantified in Supplementary Table 3. We excluded the ‘limbic’ network from this analysis due to the lack of vertices with receptor data and low signal-to-noise ratio (SNR) in functional MRI (fMRI) data.

The principal receptor gradient separates the sensory and higher cognitive networks (Fig. 5). Almost all areas of the visual and somatosensory networks had negative gradient scores. Areas in the higher cognitive networks encompassed a range of positive and negative values (Fig. 5). Taken with the above, we conclude that neurons in higher cognitive networks express more receptors.

### Gradient 2 captures 5-HT_1A_ expression across cognitive areas

**Fig. 6:**
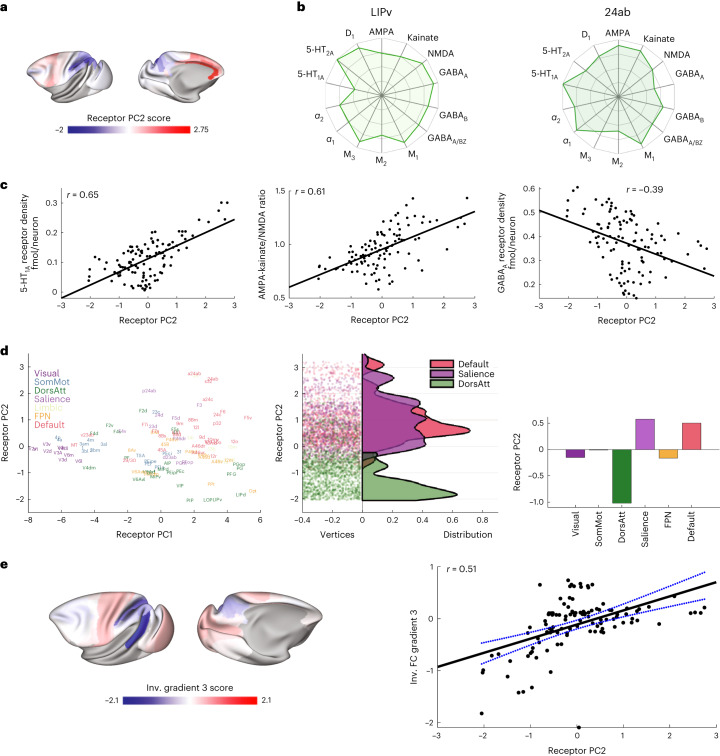
The secondary receptor gradient tracks differences in serotonin receptor densities between higher cognitive areas of cortex. **a**, Receptor PC2 (the secondary receptor gradient). **b**, Receptor fingerprints of areas 24ab and LIPv, which occupy opposing positions along the secondary gradient. The total receptor density per neuron is similar between the two areas, but there is an obvious difference in 5-HT_1A_ receptor density per neuron. For information on standard deviations, see Supplementary Table 2. **c**, Receptor PC2 was positively correlated with 5-HT_1A_ receptor density and the ratio of AMPA and kainate to NMDA receptors. Receptor PC2 was also negatively correlated with the GABA_A_ receptor density (Pearson *r* correlation). **d**, The secondary receptor gradient separates the dorsal attention network from the default mode and salience networks. Left, cognitive networks in receptor space. Middle, distribution of receptor PC2 scores within the default mode, salience and dorsal attention networks. Right, mean receptor PC2 scores for each cognitive network. Inv., inverse. **e**, Left, the third functional connectivity gradient. Right, the secondary receptor gradient was strongly correlated with the third functional connectivity gradient. The error bars in **d** (right) show the 95% upper and lower bounds of the linear fit (*r*(107) = 0.51 (range: 0.35–0.63), *P* = 0.0246 corrected for spatial autocorrelation and multiple comparisons (Bonferroni)).

The secondary receptor gradient (PC2) separates higher-order cortical areas (Fig. 6a). Parietal areas (for example, LIPv) are at one end of the gradient, whereas cingulate areas (for example, 24ab and 25) are at the other end (Fig. 6a). The receptor fingerprints reveal a striking change in the serotonin 5-HT_1A_ receptor density (Fig. 6b,c and Supplementary Fig. 7). Other receptor densities also changed along the secondary gradient, such as AMPA-kainate/NMDA ratio and GABA_A_ density (Fig. 6b,c), but not serotonin 5-HT_2A_ (Supplementary Fig. 10). The peak of serotonin 5-HT_1A_ receptor expression in the subgenual cingulate area is notable as the serotonin system and the subgenual cingulate are both targeted in treatments for depression^31^.

### Gradient 2 separates anticorrelated cognitive networks

The secondary receptor gradient has opposing values in the dorsal attention network and the default mode network (Fig. 6d). Activity in these two higher cognitive networks is often anticorrelated^32,33^. The salience network may act as a ‘switch’ between activity in these two networks^34^. We also found positive values for the secondary receptor gradient in the salience network. Gradients of functional connectivity exist in the human and macaque cortex^17,29^. This receptor gradient also resembles the third functional connectivity gradient (Fig. 6e). No other correlations between receptor and functional connectivity gradients passed this strict statistical correction. Thus, the secondary receptor gradient may suggest a serotonergic mechanism by which the cortex switches between higher cognitive states.

### 5-HT_1A_ receptor expression is conserved across species

Serotonin is involved in the pathophysiology of emotion regulation. Much of the basic research on emotion is performed in animal models. To ease the translation of findings, we compared 5-HT_1A_ expression in humans, macaques and rats. We performed this analysis on the raw receptor densities, as neuron density data were not available in all species.

**Fig. 7:**
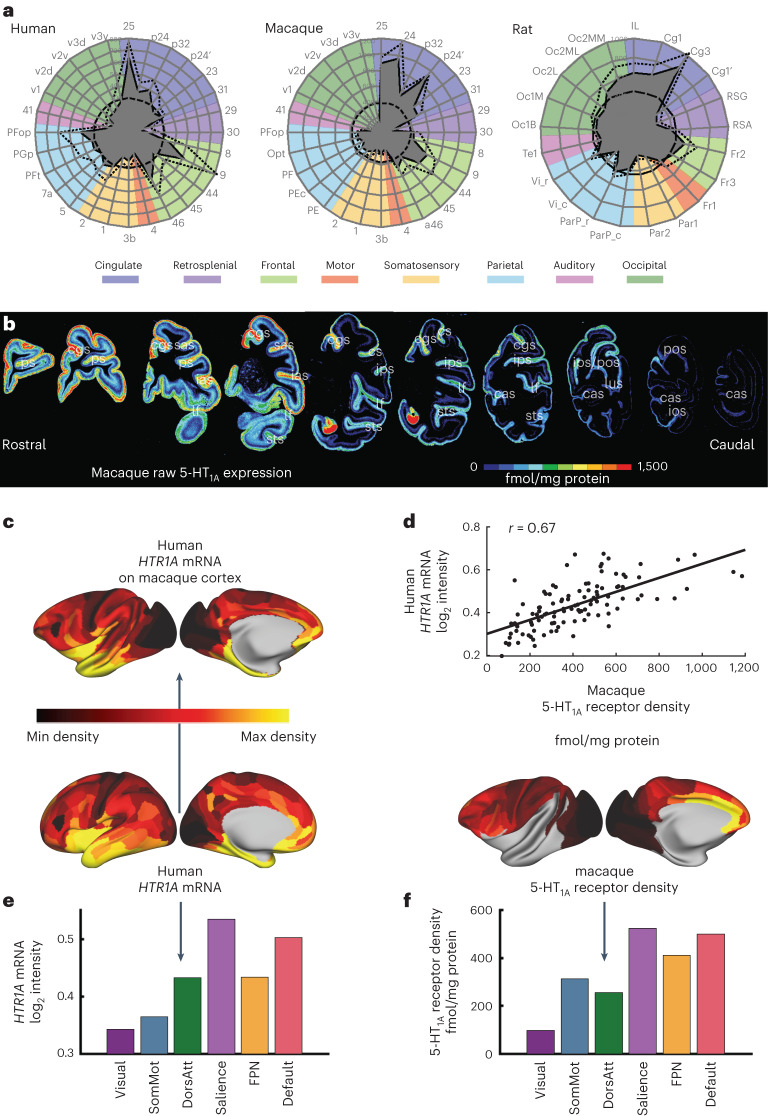
Serotonin 5-HT_1A_ receptor expression across the human, macaque and rat cortex. **a**, Density of 5-HT_1A_ receptors (in fmol mg^−^^1^ of protein) across multiple areas of human, macaque and rat cortex. The filled gray shapes indicate the mean 5-HT_1A_ receptor density within each area. Dotted lines indicate standard deviation, and the thick dashed circle indicates the average 5-HT_1A_ receptor density over all areas for the species in question. The 5-HT_1A_ receptor density is high in subcallosal or anterior cingulate in all species, although the area with peak density shifts slightly across species. **b**, The density of 5-HT_1A_ receptor expression across a single macaque cortical hemisphere. **c**, We mapped human *HTR1A* gene expression data^35^ to the human cortex and then to the macaque cortex using cross-species functional alignment. **d**, Human gene expression and macaque receptor expression for the 5-HT_1A_ receptor were positively correlated (Pearson correlation, *r*(107) = 0.66 (range: 0.54–0.76), *P* = 0.011, corrected for spatial autocorrelation). **e**,**f**, Human *HTR1A* gene expression (**e**) and macaque 5-HT_1A_ receptor expression (**f**) are expressed similarly across cognitive networks, peaking in the default mode and salience networks.

In humans, we found that 5-HT_1A_ receptor expression peaks in area 25 (subcallosal cingulate). Humans have high 5-HT_1A_ density in anterior cingulate and frontal regions and low density in motor and visual cortex (Fig. 7a). This general pattern was also true in macaque cortex and in that of the rat. However, the peak of 5-HT_1A_ receptor density shifts from area 25 to neighboring parts of anterior cingulate in the macaque. A similar shift is even more apparent in the rat. In the rat, the gradient of 5-HT_1A_ receptor density across cortex was flatter than in primates.

In the macaque brain, 5-HT_1A_ receptor density increases in the caudo-rostral and latero-medial directions (Fig. 7b). Across layers, 5-HT_1A_ receptor expression has two peaks. There is an absolute maximum in the superficial layers. A second, considerably lower peak is in layers V and VI (Supplementary Figs, 4, 9 and 10). The difference in height between both peaks is larger in sensory areas than in association areas. However, the primary visual cortex is a notable exception, because, in V1, the superficial and deep peak reach similar values. We also see this pattern in allocortical area 25 in the anterior cingulate cortex.

### 5-HT_1A_ peaks in default and salience networks across species

We investigated the relationship between receptor expression in macaques and gene expression in humans. The Allen Human Brain Atlas provides mRNA expression across six individual human brains^35^. We mapped this mRNA expression data onto a 180-area multimodal parcellation of the human cortex^1^. We used functional alignment to reverse translate this gene expression map to the macaque cortex^29^ (Fig. 7c). In the human brain, *HTR1A* (5-hydroxytryptamine receptor 1A (*Homo sapiens* (human))) encodes the 5-HT_1A_ receptor. *HTR1A* expression peaks in anterior/medial temporal cortex, insula, subcallosal/anterior cingulate and postero-medial cortex (posterior cingulate/precuneus) (Fig. 7c). We found that human *HTR1A* gene and macaque 5-HT_1A_ receptor expression are strongly correlated (Fig. 7d). Human *HTR1A* gene and macaque 5-HT_1A_ receptors are expressed similarly across cognitive networks. For both, the lowest densities are in the visual network, and the highest densities are in the default mode and salience networks (Fig. 7e,f). In contrast to 5-HT_1A_, serotonin 5-HT_2A_ receptors peak in the dorsal attention and fronto-parietal networks (Supplementary Fig. 10). Therefore, the cortical expression of serotonin 5-HT_1A_ receptors is conserved between macaques and humans. In both species, 5-HT_1A_ receptor is highest in the default mode and salience networks.

### A wide range of receptor–gene correlations across receptors

Gene expression is increasingly used as a proxy for receptor expression. However, some researchers have found weak correlations between gene and receptor expression^36^. We performed an exploratory analysis on the relationship between human gene and monkey receptor expression (Supplementary Fig. 11). We first aimed to find the upper limit on inter-species correlations. For this, we used maps of the T1w/T2w ratio and cortical thickness. These maps were acquired in a similar manner by the same laboratory in both macaques and humans^1,24^. We registered the human maps to the macaque cortex using cross-species functional alignment^29^. The cross-species correlation for the T1w/T2w map was very high (Supplementary Fig. 11; Pearson correlation, *r*(107) = 0.79 (range 0.70–0.85)). This likely represents a ceiling value for inter-species gene–receptor correlations. The correlation was moderate for cortical thickness (Pearson correlation, *r*(107) = 0.55 (range 0.40–0.67)). Some gene–receptor correlations were within or close to this range. Several correlations passed a strict correction for spatial autocorrelation^37^ (Supplementary Fig. 11; noradrenaline: α_1_-*ADRA1B* and α_2_-*ADRA2A*; serotonin: 5-HT_1A_-*HTR1A*; acetylcholine: M_3_-*CHRM3*; glutamate: AMPA-*GRIA1A*, kainate-*GRIK1*, *GRIK2* and *GRIK3*; and GABA: GABA_B_-*GABBR1* and GABA_A_-*GABRG3*). Another set of gene–receptor correlations fell just below this range and passed a less stringent threshold (*P* < 0.005, uncorrected, glutamate: AMPA-*GRIA2*, NMDA-*GRIN2B*, NMDA-*GRIN2C*, NMDA-*GRIN3A* and kainate-*GRIK5* and GABA: GABA_A_-*GABRA2*, GABA_A_-*GABRE* and GABA_A_-*GABRG1*). These gene–receptor correlations may be considered of moderate strength. It is possible that they could pass the more stringent correction if they were acquired in the same species. The remaining gene–receptor correlations, including several neuromodulatory, excitatory and inhibitory receptors, were weak. This suggests that using gene expression as a proxy for receptor expression may be appropriate for a small number of receptors, such as the 5-HT_1A_ receptor. However, for many receptors, including the dopamine D_1_ receptor, gene expression is not a good proxy for receptor expression.

### Acetylcholine and dopamine receptors vary along the cortical hierarchy

In an exploratory analysis, we compared PC3, PC4 and PC5 with each of the anatomical maps investigated above. Outside of the first two PCs, the strongest correlation was between PC4 and the cortical hierarchy (*r* = −0.50, *P* = 0.004, uncorrected; Supplementary Fig. 6h). Primary sensory areas in both visual and somatosensory cortex have high scores on PC4. This pattern is driven by a positive loading on the M_2_ receptor. This was previously identified as a potential marker of primary sensory cortex in humans^19^. There is also a negative loading on the dopamine D_1_ receptor (Supplementary Fig. 6f). This matches previous reports of D_1_ increasing strongly along the hierarchy^27^. All other correlations were weak (PC4-FC3: *r* = −0.21, *P* = 0.03, uncorrected; PC3-myelin: *r* = 0.23, *P* = 0.02, uncorrected) or not significant. Therefore, the two principal gradients of receptor expression capture the strongest spatial relationships between receptors and the structural and functional organization of the cortex.

### Receptor expression in functionally defined networks

Common brain functions activate networks of areas across the cortex. Do these different functional networks express distinct receptors? We used the NeuroQuery meta-analysis tool^38^ to define activation maps in the human cortex for functions of interest. We aligned the activation maps to the macaque cortex using cross-species functional alignment. By comparing each functional map to the first two receptor gradients, we could embed each function in the receptor space. We estimated the homology based on a recently published human–macaque homology map^29^. We also used this alignment to extract the receptor fingerprint of each functional activity map.

**Fig. 8:**
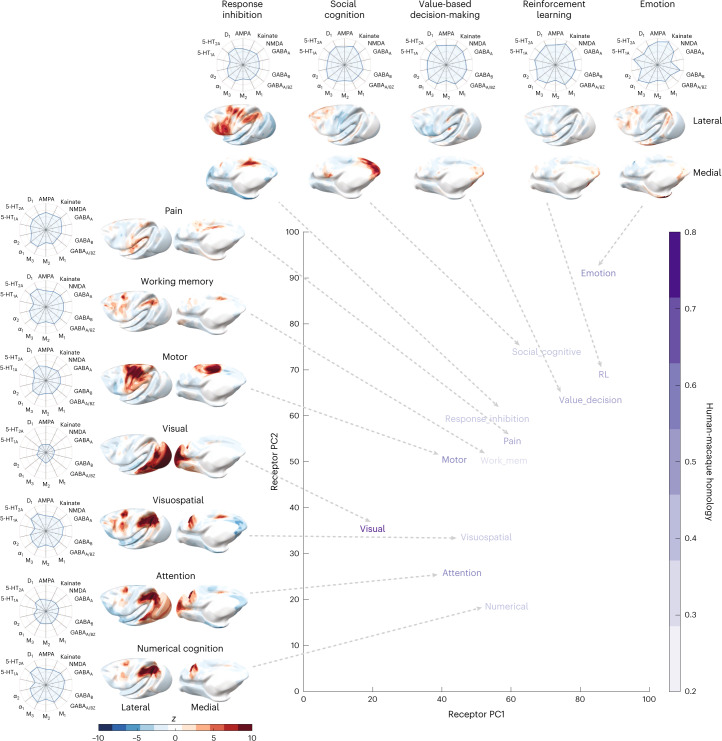
Receptor expression in functionally defined networks. Representative activation patterns for 12 functions were generated using the automatic meta-analysis software NeuroQuery. These activation maps were transformed from the human cortex to the macaque cortex using cross-species functional alignment. Each functional map is located within the two-dimensional receptor space according to the spatial overlap with the receptor gradients. The intensity of purple for each term within the plot corresponds to the estimated human–macaque homology. For each function, the receptor fingerprint plots on the outside show the average receptor density across significantly activated vertices. RL, reinforcement learning.

We found that the principal receptor gradient separates visual activity from higher cognitive activity (Fig. 8). We also observed that the second receptor gradient separates social-emotional value activity from numerical and spatial activity (Fig. 8). Activity maps of visual, motor, attention and emotion functions have a high estimated homology (Fig. 8). Activity maps of social cognitive, visuospatial, numerical, working memory and response inhibition functions have lower homology.

The receptor fingerprint for emotion stands out. The emotion activity map overlaps with areas of high 5-HT_1A_ and GABA_B_, α_1_ and kainate densities. This contrasts with numerical-spatial activity. Numerical-spatial activity maps overlap with areas that express more D_1_ and 5-HT_2A_ receptors. Therefore, this exploratory analysis suggests that gradients of receptor expression align with gradients of function along two major axes. These axes distinguish sensory-to-cognitive and emotion-to-numerical-spatial functions.

## Discussion

In this study, we measured receptor expression across the macaque cortex and uncovered general organizational principles. We discovered a principal gradient of increasing receptor expression along the cortical hierarchy. This receptor gradient separates sensory and cognitive networks. We show that the size of pyramidal cell dendrites increases with receptor expression along this gradient. In contrast, myelin and receptor expression are anticorrelated across brain areas and layers. The secondary receptor gradient is driven by the serotonin 5-HT_1A_ receptor. We show that 5-HT_1A_ receptor expression across cortex is very similar between macaques and humans. The secondary gradient segregates the dorsal attention from the default mode network and salience network. The second receptor gradient also separates activity from socio-emotional and numerical-spatial functions. This suggests a potential serotonergic basis for a switch between external and internal focus of attention and highlights the relevance of the macaque monkey as a research model.

The principal receptor gradient may help functional diversity emerge across the cortex. In the 1950s and 1960s, Sanides and Braitenberg contended that the cortex is organized in gradients^39,40^. Recently, gradients of connectivity^17,29,41^, cell types^42^, receptors^19–21^ and gene expression^43,44^ have been found. Many of these properties vary along an axis that aligns with the cortical hierarchy^26,27,45^. A puzzle is how anatomical gradients can lead to the emergence of different functions across the cortex. We chose to analyze a representative subset of receptors for classical neurotransmitters. In the human brain, the distribution of these receptors segregates cortical types and functional systems^19–21^. We discovered a principal receptor gradient in macaque cortex that increased along the hierarchy. Along the principal receptor gradient, we show that receptor expression increases by a factor of 4. We found that neurons at the top of the gradient have larger dendritic trees. They are, thus, equipped to integrate information from a greater number of sources. This may be the anatomical basis by which neurons in higher areas integrate information over longer timescales^46^ and display an increased dynamic range^47^. This large and varied receptor expression may enable neurons in higher cortical areas to act flexibly. In contrast, the neurons of early sensory cortex express relatively few receptors. This may ensure that sensory stimuli are processed reliably in different contexts. However, our analysis of dendritic properties was solely focused on the basal dendrites of layer 3 pyramidal cells^7^. This is of particular interest, as this is the site of recurrent cortical connections that allow for sustained activity^48^. This activity is thought to support many functions, including working memory and decision-making. In the future, it will be important to discover whether gradients of dendritic properties exist for pyramidal cells in other layers and for interneurons. Increased dendritic length and spine count in frontal cortex compared to V1 has been shown for the apical and basal dendrites of layer 3 pyramidal cells in the macaque but not the mouse^49^. It is possible that gradients of receptor expression and function may also differ between primates and rodents.

The second receptor gradient reveals a link between distinct treatments for depression. We found that the second receptor gradient is dominated by serotonin 5-HT_1A_ receptor expression. This resembles a pattern seen in vivo in the macaque monkey by means of receptor positron emission tomography (PET) and [^11^C]6BPWAY, a full antagonist of the 5-HT_1A_ receptor^50^. It is similar to the rostro-caudal gradient found in the human brain^19,51^. Regions scoring high on this gradient strongly overlap with areas activated during studies of emotion (Fig. 8). This gradient peaks in the cingulate cortex, with high expression in the subcallosal cingulate. This is a principal target of deep brain stimulation for treating depression^31^. Serotonin release may be reduced in patients with depression. Interestingly, deep brain stimulation of the subcallosal cingulate and selective serotonin reuptake inhibitors (SSRIs) have almost identical effects on cerebral blood flow^31,52,53^. SSRIs increase the activation of 5-HT_1A_ receptors. They act to reduce neural activity around the subcallosal cingulate. This counteracts the increased glucose metabolism seen in patients with depression in this area^54^. Our analysis of 5-HT_1A_ receptor expression suggests that subcallosal cingulate stimulation and SSRIs target the same brain network.

The macaque may be a promising animal model for serotonergic functions and related disorders. Here, using in vitro autoradiography, we found that 5-HT_1A_ receptor density in the macaque is very similar to the *HTR1A* gene expression and receptor expression in humans (Fig. 7 and Supplementary Fig. 7). Gene expression is not always a good predictor of receptor expression. However, a previous study (using PET) found that, in humans, 5-HT_1A_ receptor and gene expression are highly correlated^55^. Here, 5-HT_1A_ had one of the highest gene–receptor correlations of all receptors (Supplementary Fig. 11). This is consistent with a contemporaneous analysis of gene–receptor correlations in the human brain by other authors^36^. In the rat, 5-HT_1A_ receptor expression also peaked in the cingulate cortex. However, the gradient of expression was flatter than in the macaque or human brain. Notably, in the rat, the laminar receptor expression pattern differs from that observed in human and macaque cortex^6^. Receptors for serotonin and other neuromodulators may also be expressed on different cell types between rodents and primates^56–58^. Recognizing differences in serotonin receptor expression across species may be important when interpreting animal models of serotonergic functions and disorders.

The secondary receptor gradient separates the dorsal attention network from the default mode network and salience network. Association cortex can be divided into four networks (dorsal attention, salience, frontoparietal and default). These networks each occupy parts of the frontal, parietal and temporal lobes. In several patches of cortex, they appear in a consistent order^17^. Among the higher cognitive networks, the dorsal attention network lies closest to sensory areas. The dorsal attention network is active when attention is focused on external stimuli^59^. The default mode network lies farthest away from sensory areas. The default mode network is active when attention is not focused on the external world. This includes during autobiographical memory or imagination^60^. Activity in these two networks is often anticorrelated^32,33^, in line with their opposing roles in cognition. The frontoparietal network (also known as the multiple demand system, the cognitive control network and the central executive network) lies anatomically between these two networks. The frontoparietal network may couple with either of the other two networks, depending on task demands^61^. In line with this role, we found that the frontoparietal network lies between the dorsal attention and default mode networks along the secondary receptor gradient (Fig. 6).

Serotonin and noradrenaline release may shift the brain from internally focused to externally focused attention states. The mechanism of antagonism between the dorsal attention network and the default mode network is unknown. It could result from long-range projections to inhibitory neurons^62^. Here, we show that serotonin release should engage 5-HT_1A_ receptors in the default mode and salience networks. The noradrenaline α1 receptor similarly peaks in these networks. The salience network can switch the brain from default mode network to frontoparietal and dorsal attention network-dominated activity^34^. Some stimuli, such as surprising stimuli, activate the salience network and also induce serotonin and noradrenaline release^34,63,64^. This suggests a functional link between these systems that supports the anatomical results found here. The 5-HT_1A_ receptor has a high affinity for serotonin. For this reason, it normally dominates cortical serotonin processing. In contrast, massive serotonin release under extreme conditions engages 5-HT_2A_ receptors^65^. These are events when attention needs to be rapidly shifted to external stimuli. We show that the excitatory effects of the 5-HT_2A_ receptors may complement the 5-HT_1A_ effects by exciting the dorsal attention and frontoparietal networks (Supplementary Fig. 11). Therefore, serotonin and noradrenaline release may shift activity between relatively stable states. This is compatible with recent findings that genes for neuromodulatory receptors are expressed at cortical locations that may affect the flow of brain states over time^66^. This suggests a potential neuromodulatory mechanism by which the brain may shift activity between cardinal cognitive networks.

The high neuron density in V1 underlies its high receptor expression. A gradient of receptor expression was recently discovered in the human cortex^19–21^. The human receptor gradient resembles the principal receptor gradient of the macaque brain. An advantage of studying the macaque cortex is that it allows for comparison with gold standard invasive anatomy data. For example, comprehensive maps of neuron density are not currently available for the human^8^. We compared the receptor and neuron density data. This revealed that several receptors are highly expressed in V1 because of the high neuron density in that area. The receptor maps that we provide are only a snapshot in time. In the future, much remains to be discovered, including the variation by sex, pathological changes associated with brain disorders and changes across time.

The present study provides insights into the relationships between the densities of 14 receptors from six classical neurotransmitter systems and cortical hierarchy in the macaque monkey brain. Across these receptors, some methodological considerations must be considered because, despite decades of research, not all currently available ligands are able to completely segregate different receptor types (for example, D_1_ and D_5_ receptors; Supplementary Table 4). Interestingly, the NMDA receptor antagonist [^3^H]MK-801 was found to inhibit human nicotinic acetylcholine receptors by blocking the open pore^67,68^. Because the nicotinic receptor channel requires acetylcholine to open, and all endogenous substances were removed during the pre-incubation step of the binding protocol, it is highly unlikely that the binding sites labeled here with [^3^H]MK-801 include the nicotinic cholinergic receptor. Future studies will be necessary to develop, characterize and quantify the distribution of more specific radioligands for all receptor types analyzed here.

Recent developments in large-scale recordings have highlighted the distributed nature of cognitive functions. However, theoretical understanding of how cortical activity patterns enable function remains limited. The receptor data presented here, along with connectivity data^3^, can provide an anatomical basis for large-scale models and theories of brain function^27,69–74^. Future large-scale theories of brain function may reveal how flexible higher cognition emerges along the principal receptor gradient.

## Methods

### In vitro receptor autoradiography

Quantitative in vitro receptor autoradiography was applied to label 14 neurotransmitter receptors in three male *Macaca fascicularis* brains (7.3 ± 0.6 years old; body weight 6 ± 0.8 kg) obtained from Covance Preclinical Services, where they were housed and used as control animals for pharmaceutical studies performed in compliance with legal requirements. Serotonin 5-HT_1A_ receptor densities were also quantified in six adult male Lewis rat brains from the Central Animal Facility of the Hannover Medical School. Animal experimental procedures and husbandry had the approval of the respective Institutional Animal Care and Use Committee and were carried out in accordance with the European Council Directive of 2010. Human data used here were previously published^19,76,77^ and come from the brains of five donors (three males, 76 ± 3 years old) without a history of neurological or psychiatric diseases. No statistical methods were used to pre-determine sample sizes, but our sample sizes are similar to those reported in previous publications^12,19,22,23,76,77^.

Monkeys were sacrificed by an intravenous lethal dose of sodium pentobarbital, and brains were removed immediately from the skull. Brain stem and cerebellum were dissected off in close proximity to the cerebral peduncles, and hemispheres were separated into a rostral and a caudal block by a cut in the coronal plane of sectioning within the central sulcus. Rats were decapitated under ketamine–xylazine narcosis, and the brains were removed immediately from the skull. Brain tissue was shock frozen in isopentane at −40 °C to −50 °C.

Brain tissue was serially sectioned in the coronal plane (section thickness 20 µm) using a cryostat microtome (Leica, CM3050S). Sections were thaw mounted on gelatine-coated slides, sorted into 22 parallel series and freeze dried overnight. Alternating series of sections were processed for visualization of receptors according to previously published protocols, which were also established for the macaque brain^78,79^ (Supplementary Table 4), cell bodies^80^ or myelin^81^, so that there was a gap of ~800 µm between two acquired sections for a given receptor type or histological staining.

Receptor binding protocols encompass a pre-incubation to rehydrate sections, a main incubation with a tritiated ligand in the presence of or without a non-labeled displacer and a final rinsing step to terminate binding (Supplementary Table 4). Incubation with the tritiated ligand alone demonstrates total binding; incubation in combination with the displacer reveals the proportion of non-specific binding sites. Specific binding is the difference between total and non-specific binding and was less than 5% of the total binding. Thus, total binding is considered to be equivalent of specific binding^78^. Sections were exposed together with standards of known radioactivity against tritium-sensitive films (Hyperfilm, Amersham) for 4–18 weeks depending on the receptor type.

Ensuing autoradiographs were processed by densitometry with a video-based image analyzing technique (for methodical details, see refs. ^25,78^). In short, autoradiographs were digitized as 8-bit images. Gray values in the images of the standards were used to compute a calibration curve indicating the relationship between gray values in an autoradiograph and binding site concentrations in femtomole per milligram (fmol mg^−1^) of protein. Concentrations of radioactivity (*R*, counts per minute) in each standard, which had been calibrated against brain tissue homogenate, were converted to binding site concentrations (*C*_b_, fmol mg^−1^ of protein) using:

$${C}_{{\mathrm{b}}}=\frac{R}{E\times B\times {W}_{{\mathrm{b}}}\times {S}_{{\mathrm{a}}}}\times \frac{{K}_{{\mathrm{D}}}+L}{L}$$

where *E* is efficiency of scintillation counter used to measure concentration of radioactivity in brain tissue homogenate (Hidex, 300SL); *B* is a constant representing number of decays per unit of time and radioactivity (Ci min^−1^); W_b_ is protein weight of a standard (mg); *S*_a_ is specific activity of the ligand (Ci mmol^−1^); *K*_D_ is the dissociation constant of the ligand (nM); and *L* is concentration of the ligand during incubation (nM, measured by liquid scintillation counting using same counter as for brain tissue homogenate). The ensuing calibration curve is used to linearize the autoradiographs—that is, to convert the gray value of each pixel into a binding site concentration in fmol mg^−1^ of protein.

Cortical areas were identified in the cell-body-stained sections based on previously published criteria for the rat^82^ and macaque (Visual^23^; Parietal^22,79,83^ and Motor^12^; Cingulate^84^, Prefrontal and Orbitofrontal^85–87^) brains, and borders were transferred to the neighboring sections processed for receptor autoradiography. The mean receptor density for each area was determined by density profiles extracted vertical to the cortical surface over a series of 3–5 linearized autoradiographs per receptor type, area and brain using MATLAB-based in-house software^25,78^. Specifically, densities were extracted from three sections spread throughout areas of the rat brain or small areas of the macaque brain (for example, area 25) and from five sections for larger areas of the macaque brain (for example, p24).

### Surface representation of cyto-architectonic and receptor-architectonic atlas and receptor data

In total, 109 cortical areas were defined in the macaque monkey brain based on their cyto-architecture and receptor-architecture, as described above. We call this parcellation the Julich Brain Macaque Maps (https://search.kg.ebrains.eu/instances/e39a0407-a98a-480e-9c63-4a2225ddfbe4). The location and extent of the cortical areas were delineated in the three-dimensional space of the Yerkes19 surface^24^ by L.R., M.N. and N.P.G. using the connectome workbench software (https://www.humanconnectome.org/software/connectome-workbench) by carefully aligning boundaries to macro-anatomical landmarks identified using the cyto-architecture. The location of all regions on the Yerkes19 surface were independently checked and verified by M.N., S.F.W., L.R. and N.P.G. Additionally, the mean receptor densities of all 14 receptor types have been projected onto the corresponding area on the Yerkes19 surface for visualization.

### Surface representation of neural density data

Collins et al.^8^ measured neural density across the macaque cortex using isotropic fractionation. Those authors studied the brain of a 4-month-old macaque monkey. Adult neural density is already present at this age^88^. In that article, the cortex is presented as a flat map divided into sections (that is, the Vanderbilt sections; their Fig. 2 and Supplementary Fig. 6). We used these maps, along with several sulcal and areal landmarks (their Fig. 2), to estimate the location of each cortical section. The landmarks included the following sulci: calcarine sulcus, lunate sulcus, intraoccipital sulcus, occipitotemporal sulcus, intraparietal sulcus, cingulate sulcus, lateral sulcus, arcuate sulcus and principal sulcus and the following brain areas: V1, V2, V4, DM, LIP, MIP, VIP, AIP, MT, A1, parabelt, insula, 5a, 2, 1, 3b, M1, PMv, PMd, FEF and SMA. We then transferred the locations of the sections onto the Yerkes19 surface.

This was performed by S.F.W. and independently verified by L.R., M.N. and N.P.G. This allowed us to estimate the neural density in each of the 109 areas of the Julich Brain Macaque Maps. Several of the brain areas in the Julich Brain Macaque Maps overlapped with two or more of the Vanderbilt sections. In these cases, we estimated the neural density in each Julich Brain Macaque Maps area according to the degree of overlap with each Vanderbilt section.

The neuron density data were originally in units of neurons per gram, and the receptor density data were in fmol mg^−^^1^ of protein. To estimate the receptor density in fmol per neuron, we used the previously reported figure that 8% of brain tissue is protein^89^. This amounts to multiplying by a constant and does not affect the calculation of the gradients via PCA or the correlations with other maps.

### Receptor gradients

To identify the receptor gradients, we *z*-scored the receptors-per-neuron data and performed a PCA. *z*-scoring ensured that high-density receptors would not dominate the PCs.

### Cortical hierarchy and retrograde tracing data

The cortical connectivity data were obtained from Henry Kennedy (Lyon, France) and are available at https://core-nets.org. The retrograde tracing data were obtained by injections into 40 cortical regions^27,90^. This was performed using consistent methods in the same laboratory. We recently estimated the cortical hierarchy using these data, based on the laminar patterns of connections^27^. The parcellation for this connectivity data is already available on the Yerkes19 surface. This parcellation is known in BALSA as the M132 atlas. We used this to fill in hierarchy values on the surface. We estimated the receptor gradients within each area of the M132 Atlas. Gradient values were calculated based on the overlap with each area of the Julich Brain Macaque Maps.

### Surface representation of dendritic data

Elston et al. measured dendritic tree length and spine density across the macaque cortex. In our study, we analyzed the data for layer 3 pyramidal neurons, for which most data are available. We mapped the injection sites onto the Yerkes19 template. Borders for injection sites in the series of papers by Elston et al. were drawn on the Yerkes19 template by S.F.W. All identified injection sites on the cortical surface were independently verified by M.N., L.R. and N.P.G. Direct comparison with the hand-drawn maps was possible for areas V1, V2, MT, LIPv, 7a, V4, TEO, STP, IT, ant. cing., post. cing, TEpd, 12vl, A1, 3b, 4, 5, 6, 7b, 9, 13, 46 and 7m^91–99^. The following references were also used to identify the locations of injection sites on the Yerkes19 surface. Areas 10, 11 and 12 (ref. ^100^) were described with reference to ref. ^101^. The injection in area TEa, as described in ref. ^102^, used the maps in Seltzer and Pandya^103^ for area definition. We used these maps to approximate the injection location. Area STP was identified with the corresponding region STPp in the atlas of Felleman and Van Essen^104^. Area FEF was identified according to the description in ref. ^105^. It is described as lying on the anterior bank of the medial aspect of the arcuate sulcus. The receptor PC score was averaged within all vertices in each injection site. This allowed us to compare dendritic and receptor data.

### Cortical T1w/T2w data

The T1w/T2w data were acquired by Donahue et al.^24^ and were downloaded from the BALSA neuroimaging website (https://balsa.wustl.edu/study/W336). To compare the T1w/T2w data with the receptor data, we simply averaged the T1w/T2w signal within each of the 109 areas of the Julich Brain Macaque Maps.

### Cross-species functional alignment and functional connectivity data

We used cross-species functional alignment to compare macaque and human data. This method relies on three major steps: (1) construct a cross-species joint similarity matrix; (2) based on the joint similarity matrix, calculate matching gradients of functional connectivity across species; and (3) use the functional connectivity gradients as input to multimodal surface matching (MSM), to create a vertex-to-vertex mapping across species. For details of the method, see Xu et al.^29^; for a similar approach, see Mars et al.^106^. The original macaque data were from Oxford, obtained via PRIME-DE. The original human data were from the Human Connectome Project (HCP).

Xu et al.^29^ transferred the cognitive networks defined in Yeo et al.^30^ from human to macaque using this cross-species functional alignment^29^. We used this human-to-monkey mapping to identify the receptor expression across cognitive networks. We excluded the limbic network from analysis. This is due to a lack of receptor data and a very low SNR in fMRI data. We also used this method to compare human gene expression and functional activation to macaque receptor data.

### Human gene expression data

Human gene expression data were downloaded from the Allen Human Brain Atlas^35^. We analyzed data from hundreds of microarray samples across the left cortical hemispheres of six donors (five males, one female, age 24–55 years).

We performed the following steps to process the gene expression data.

1. Remove probes without a valid Entrez ID
2. For each subject
  - Extract left hemisphere cortical samples in native MRI space from the SampleAnnot file
  - Register samples to the native FreeSurfer conformed space
  - Map samples to the cortical ribbon, using the manually curated individual cortical surface reconstructions of Vincent Beliveau and Melanie Ganz (https://surfer.nmr.mgh.harvard.edu/fswiki/AllenBrainAtlas)^55^
    (i) Remove samples >2 mm from the cortical ribbon
    (ii) Move samples on edge of cortical ribbon to closest cortical voxel containing no samples
  - Map samples from volume to individual surface
  - Register native and fs_LR (HCP) surfaces via the FSaverage surface, using surface-based alignment. This is likely preferable to volume-based alignment due to the nonlinear distortions that are common with postmortem brains.
  - Move each sample to closest HCP cortical vertex containing no samples
  - Remove samples from medial wall
3. Remove samples with exceptionally low inter-areal similarity^43^
4. *z*-score across probes, within samples
5. Map cortical samples to cortical areas, from the HCP MMP parcellation^1^
6. Remove probes that do not have above-threshold expression in at least 40% of cortical areas (parcels)^43^
7. Account for inter-individual differences using scaled robust sigmoid normalization^107,108^
8. Map significant probes to cortical areas
  - For each area and probe
    (i) Check if there are any samples within an area that show significant expression for that probe
      1. If there are, calculate the average expression of that probe across all the significant samples within the area
      2. Otherwise, for each vertex in the area, find the closest sample with significant expression of that probe. Then, calculate the average of the closest samples (one per vertex) to estimate the expression for the area^43^.
9. Create the gene expression by cortical area matrix. For each gene assessed via multiple probes, we selected the most representative probe, as follows^43^. Where two probes were available, we selected the probe with the maximum gene expression variance across cortical areas. If three or more probes were available, we selected the probe with the highest average correlation (of expression across areas) with the other probes.

Our code for pre-processing and analyzing gene expression data on the cortical surface is available. The code also enables easy visualization of gene expression data on the cortical surface. Lastly, it allows for easy correlation of gene expression data with various other anatomical and functional maps. Access the code on github (https://github.com/seanfw/genemapper).

### Receptor expression within functional activity maps from NeuroQuery

We used a human meta-analysis database to explore potential receptor expression–function associations. We first identified 12 representative cognitive topics. These included basic sensorimotor functions (for example, visual, pain and motor) and higher-order functions (for example, attention, emotion, reinforcement learning and working memory). We performed a decoding analysis based on the recently published meta-analysis database NeuroQuery^38^. We then generated the likelihood *z*-map in human Montreal Neurological Institute (MNI) volume space. Next, we mapped each of the likelihood *z*-maps from the MNI volume space to the FreeSurfer fsaverage surface. This was performed using the RF-ANTs (that is, ANTs-based registration fusion) tool^109^. After that, *z*-maps were further transformed to HCP fsLR surface using the HCP tool wb_command. *z*-maps were then projected to the macaque Yerkes19 template space using cross-species functional alignment^29^. We then binarized the likelihood *z*-maps (thresholded at *z* > 3.1). This enabled us to locate the significant brain areas associated with each cognitive topic. Finally, we examined the association between cognitive topics and receptor expression patterns. We did this by measuring the overlap (that is, Dice coefficient) of the binarized maps with 20 percentile bins of receptor gradient maps in macaque space^17^. Note that the functional activation map is driven from the human meta-analysis database. Thus, we used the human–macaque homology score to weight the cognition terms of interest. For each of the 12 functions, we extracted the average receptor expression across all vertices with significant functional activation (*z* > 3.1).

### Statistical analysis

The mean receptor expression across three brains was used for most analysis. We analyzed variation in receptor expression across 109 regions of these brains. Pearson correlations were performed between the receptor PCs and several other anatomical and functional maps. Data distribution was assumed to be normal, but this was not formally tested. Similar results were obtained using equivalent non-parametric tests. We provide the two-tailed *P* value, adjusted to account for the spatial autocorrelation of the data^37^. These spatial *P* values were then Bonferroni corrected based on the number of correlations between receptor gradients and structural or functional maps. No animals or data points were excluded from the analyses for any reason.

We defined the gradient dependence of PCs on receptor types as follows.

We first compute the singular value decomposition of the normalized receptor data matrix. The data were normalized by removing the mean from each column and then dividing each column entry by that column’s standard deviation.

$${X}_{{\mathrm{norm}}}=US{V}^{{\mathrm{T}}}$$

The representation of the data in the PC space is given by

$$Z=US$$

In the singular value decomposition, *X*_norm_ = *USV*^T^. *S* is a diagonal matrix, with the singular values of *X*_norm_ along the diagonal in decreasing order. *U* is an orthonormal matrix whose columns are the left singular vectors of *X*_norm_. To reconstruct the original normalized data, we can multiply *Z* by *V*^T^. *V* is an orthonormal matrix whose columns are the right singular vectors of *X*_norm_. T here denotes the transpose.

$${X}_{{\mathrm{norm}}}=Z{V}^{{\mathrm{T}}}$$

To reconstruct the data with the first (*n* − 1) PCs removed (*X*^PC*n*+^), we can calculate

$${X}^{{\mathrm{PC}}n+}={Z}_{n+}{V}_{n+}^{{\mathrm{T}}}$$

where *Z*_*n+*_ and *V*_*n+*_ are *Z* and *V* with the first (*n* − 1) columns removed.

For each PC *n*, we repeated the following procedure. We removed each receptor type *r*, one at a time, from the dataset *X*^PC*n*+^ with (*n* − 1) PCs removed, to obtain a new reduced dataset *X*_*r−*_^PC*n*+^. This is similar in spirit to earlier leave-one-receptor-out analyses^110^. We then calculated the projections (**Z**_1,r−_^PC*n*+^) of the data onto the first PC of this reduced dataset. Note that if we do not remove any receptors from the dataset, then the first PC of *X*^PC*n*+^ corresponds to the *n*th PC of the original dataset *X*_norm_. For each PC and receptor, we calculated the Pearson correlation between the projection of the data onto the original PC and the new projection:

$$\rho_{{{\mathbf{Z}_{n}}},{{{\mathbf{Z}_{1,r-}}}}^{{\mathrm{PC}}n+}} = \frac{{\mathrm{cov}}({{\mathbf{Z}_{n}}},{{\mathbf{Z}_{1,r-}}}^{{\mathrm{PC}}n+})}{\sigma_{{{\mathbf{Z}_{n}}}}\sigma_{{{\mathbf{Z}_{1,r-}}}^{{\mathrm{PC}}n+}}}$$

where cov is the covariance and *σ* is the standard deviation. The gradient dependence *g* is defined as

$$g=1-\rho_{({{\mathbf{Z}_{n}}},{{{\mathbf{Z}_{1,r-}}}}^{{\mathrm{PC}}n+})}^{2},$$

where squaring accounts for the arbitrary signs of PCs.

### Reporting Summary

Further information on research design is available in the Nature Portfolio Reporting Summary linked to this article.

## Online content

Any methods, additional references, Nature Portfolio reporting summaries, source data, extended data, supplementary information, acknowledgements, peer review information; details of author contributions and competing interests; and statements of data and code availability are available at 10.1038/s41593-023-01351-2.

## Supplementary information

Supplementary Information Supplementary Figs. 1–11 and Tables 1–4.

Reporting Summary

## Data Availability

Data are available in table format in Supplementary Tables 1 and 2 and are available on the BALSA neuroimaging repository (study ID: P2Nql, https://balsa.wustl.edu/study/P2Nql) and the Human Brain Project platform EBRAINS (10.25493/5HK3-S8M).

## Code availability

Code used for data integration and analysis in this study is available on GitHub (github.com/seanfw/macaque-receptor-gradients and github.com/seanfw/genemapper).
