# Supplementary material for: Gradients of neurotransmitter receptor expression in the macaque cortex
Source: Nat Neurosci. 2023 Jun 19;26(7):1281–94. doi: 10.1038/s41593-023-01351-2 (PMC10322721; doi:10.1038/s41593-023-01351-2)
Supplement: Supplementary file 1 — Supplementary Figs. 1–11 and Tables 1–4. [file 41593_2023_1351_MOESM1_ESM.pdf]

nature neuroscience

Resource https://doi.org/10.1038/s41593-023-01351-2

Gradients of neurotransmitter receptor expression in the macaque cortex

In the format provided by the authors and unedited

Gradients of receptor expression in the macaque cortex

Sean Froudist-Walsh, Ting Xu, Meiqi Niu, Lucija Rapan, Ling Zhao, Daniel S. Margulies, Karl Zilles, Xiao-Jing Wang, Nicola Palomero-Gallagher

SUPPLEMENTARY MATERIAL

Figure S1. Consistent receptor labelling within brain regions. A) within subjects, across sections and B) across subjects

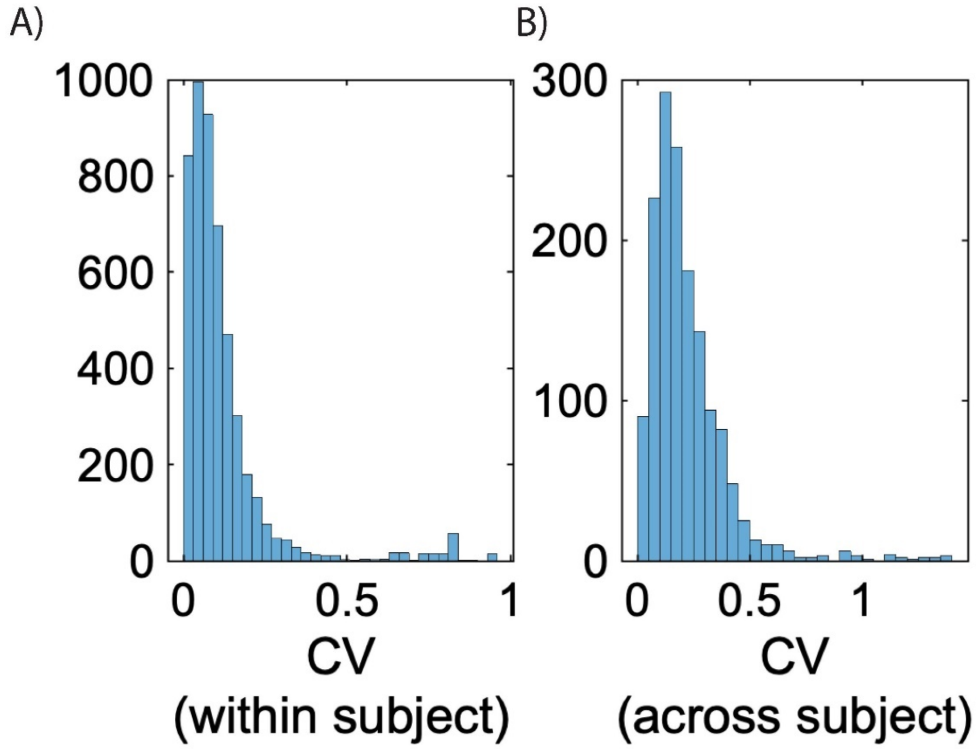

Figure S2. Algorithm-based detection of areal borders of LIPd in exemplary receptor autoradiographs. A) The receptor α_1 is shown as an example. **i)** This shows automatic labeling of the position of the statistically defined borders of LIPd (at positions 28 and 101). **ii)** The Mahalanobis distances between neighboring blocks of 22 profiles; significant maxima occurred at profile positions 28 and 101, which identify the Opt/LIPd and LIPd/LIPv borders, respectively. **iii)** The significant maxima of varying block sizes (ranging from 12 to 24). This indicates consistently occurring borders between cortical areas at profile positions 28 and 101. **iv)** The position of borders as defined by visual inspection is indicated by white bold line in the corresponding pseudocolor coded autoradiograph. The same border is shown below on corresponding sections of B) α_2 and C) 5-HT_{1A} receptors. Note the close resemblance of the position of the borders in different receptor sections.

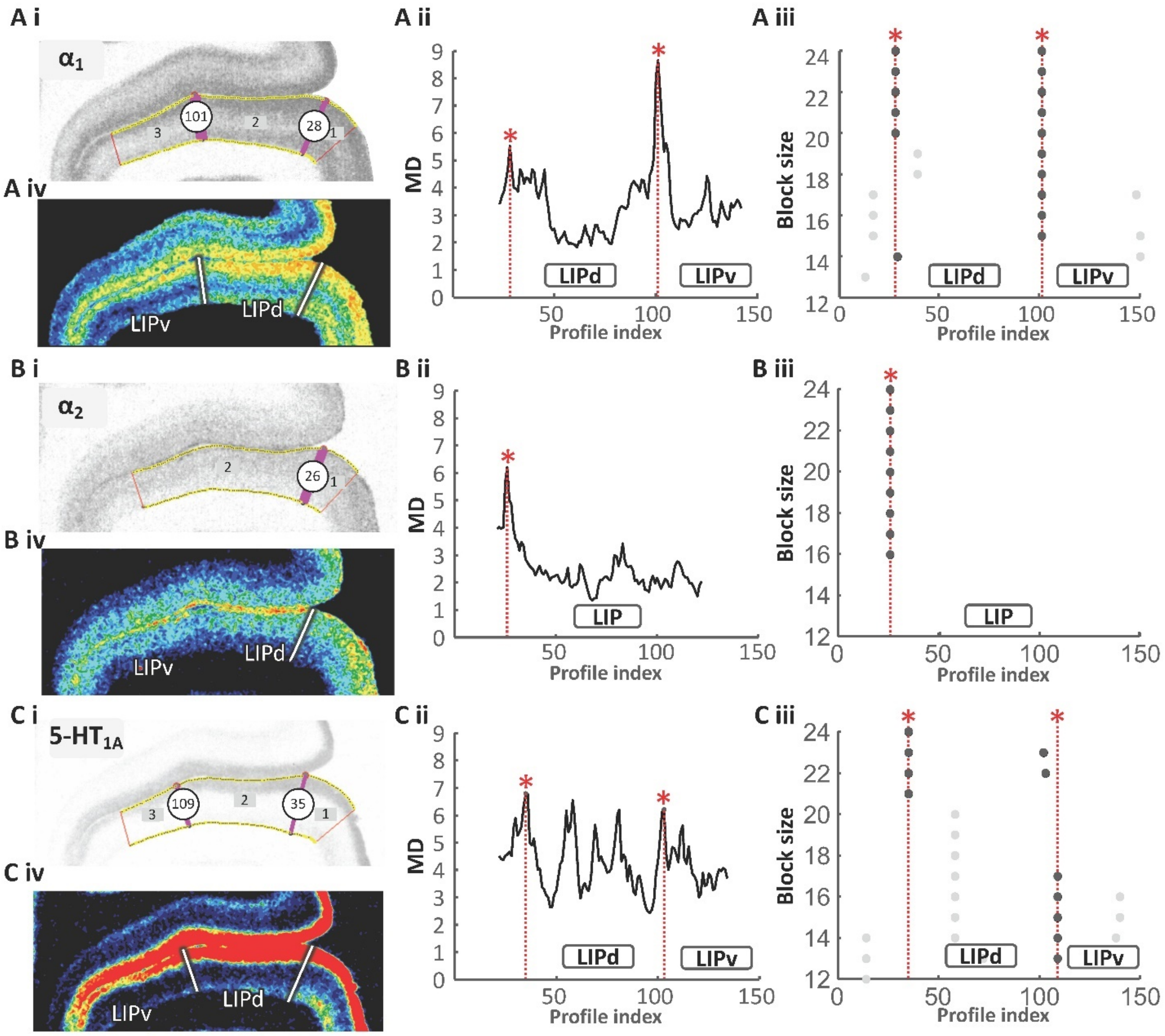

Figure S3. The density of 14 receptors across macaque cortex. A) The raw receptor density of 14 receptor types assessed with in-vitro receptor autoradiography. B) The ratio of densities for neurons and each receptor type, formed by the area with maximum density divided by the area with minimal density.

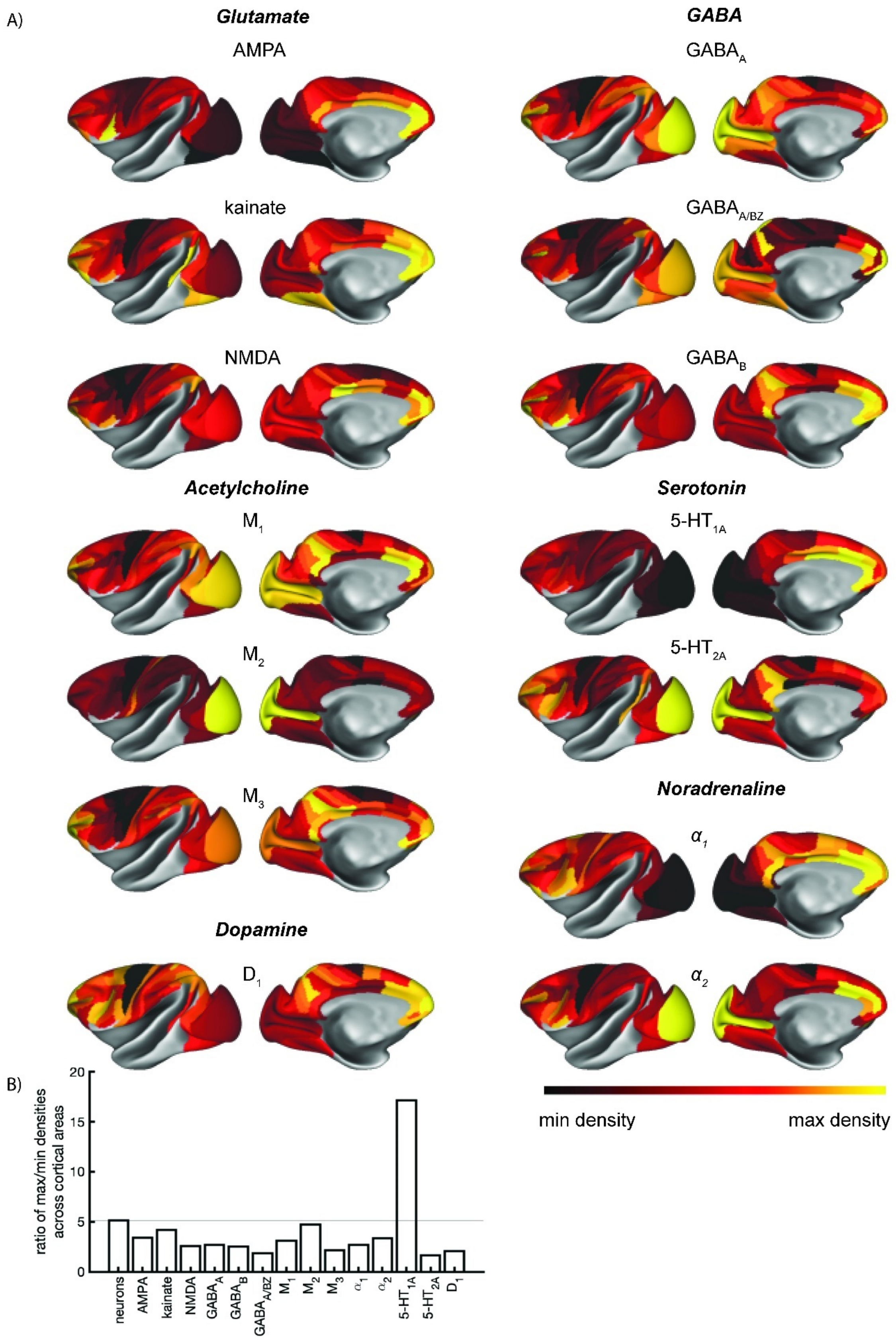

Figure S4. Polar plots of receptor expression per neuron across cortical areas. Mean expression of a given receptor within an area is shown by the thick continuous line. Mean plus standard deviation is shown by the dashed line, and average expression across all areas is shown by the thin continuous colored line. Colors code for different receptor types.

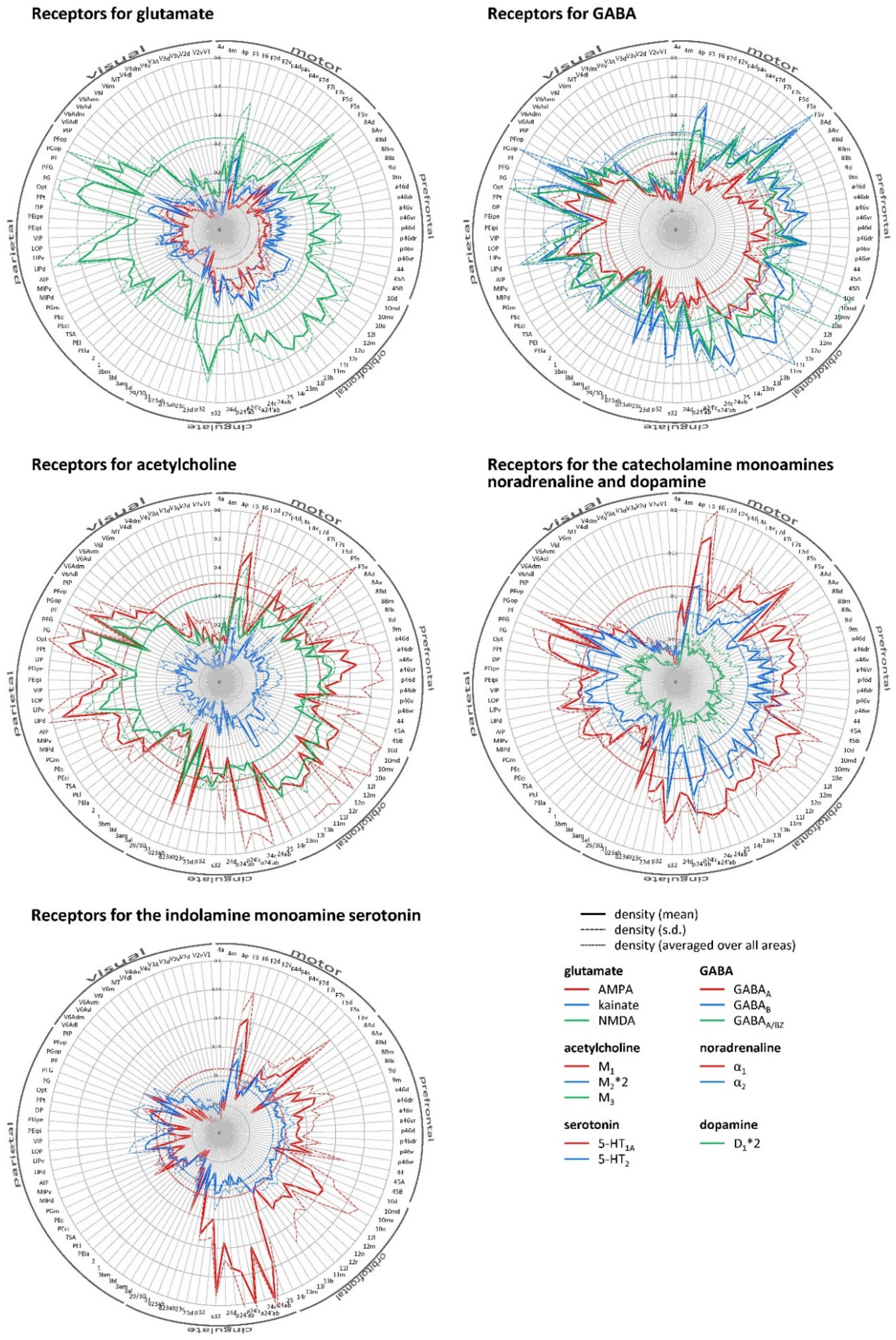

Figure S5. Principal components of the raw receptor data. A) The first five principal components of the raw receptor data. B) The strength of correlation between each of the top 5 principal components in the raw and neuron-density corrected data. The absolute value of the Pearson correlation coefficient is shown, as the sign of principal components is arbitrary. Note that each principal component of the raw receptor data is most strongly correlated with the corresponding principal component of the neuron-density corrected data. C) The projection of brain regions onto the first two principal components of the raw receptor data ('raw receptor space'). Brain regions clustered into rough anatomo-functional groups in receptor space. D) The principal component coefficients for each receptor type contributing to principal components 1-5 of the raw receptor data.

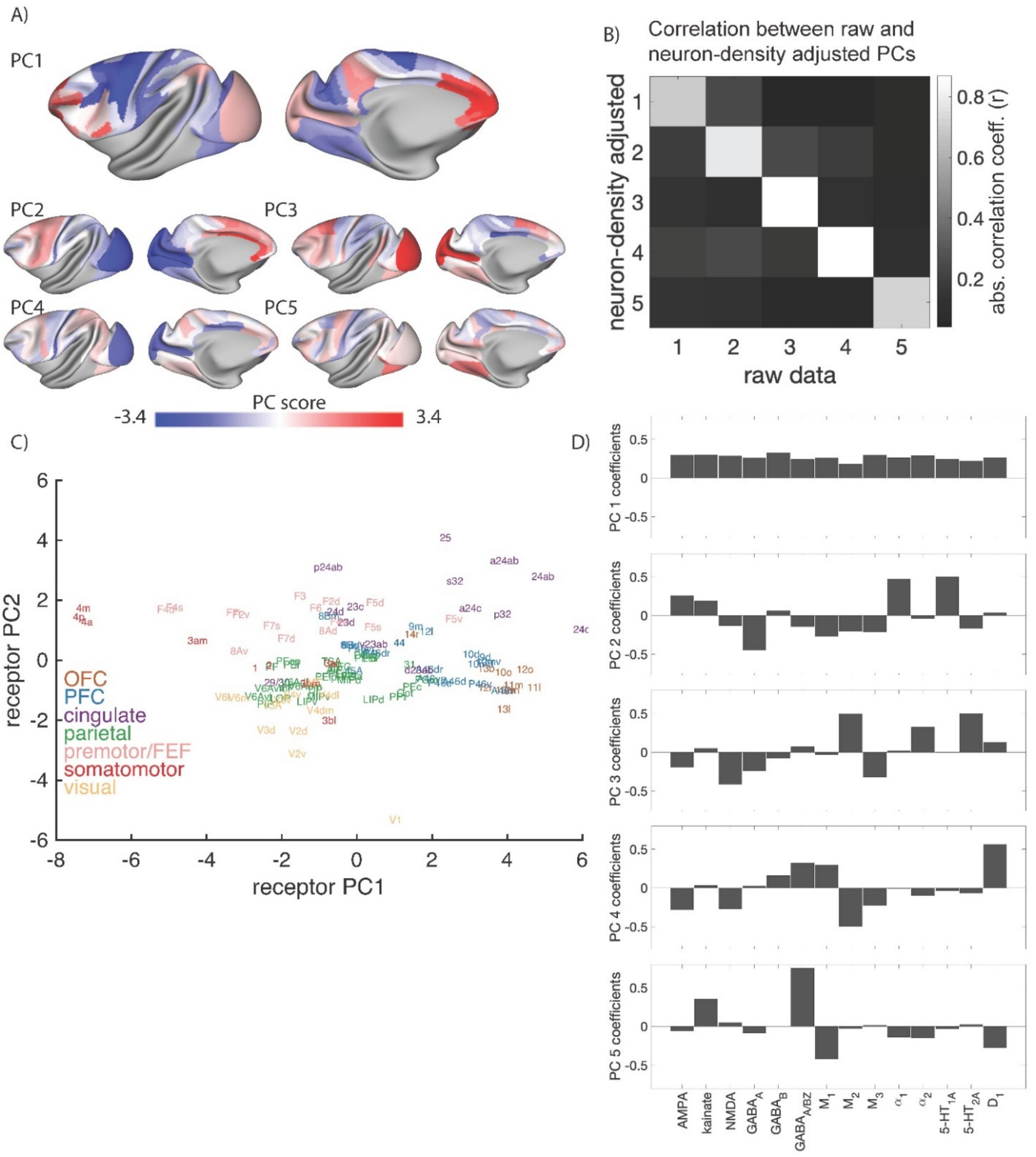

Figure S6. Principal components of the receptor data. A) The variance explained, and B) cumulative variance explained of each principal component. The top five principal components explained 95% of the variance in the data. C-G) The principal component coefficients for each receptor type contributing to principal components 1-5. H) The Pearson's r for correlations between each principal component of the receptor data and each of the structural and functional maps of brain organization. Thresholded at $p<0.05$. FC = functional connectivity gradient. Note that different numbers of data points were available for different maps.

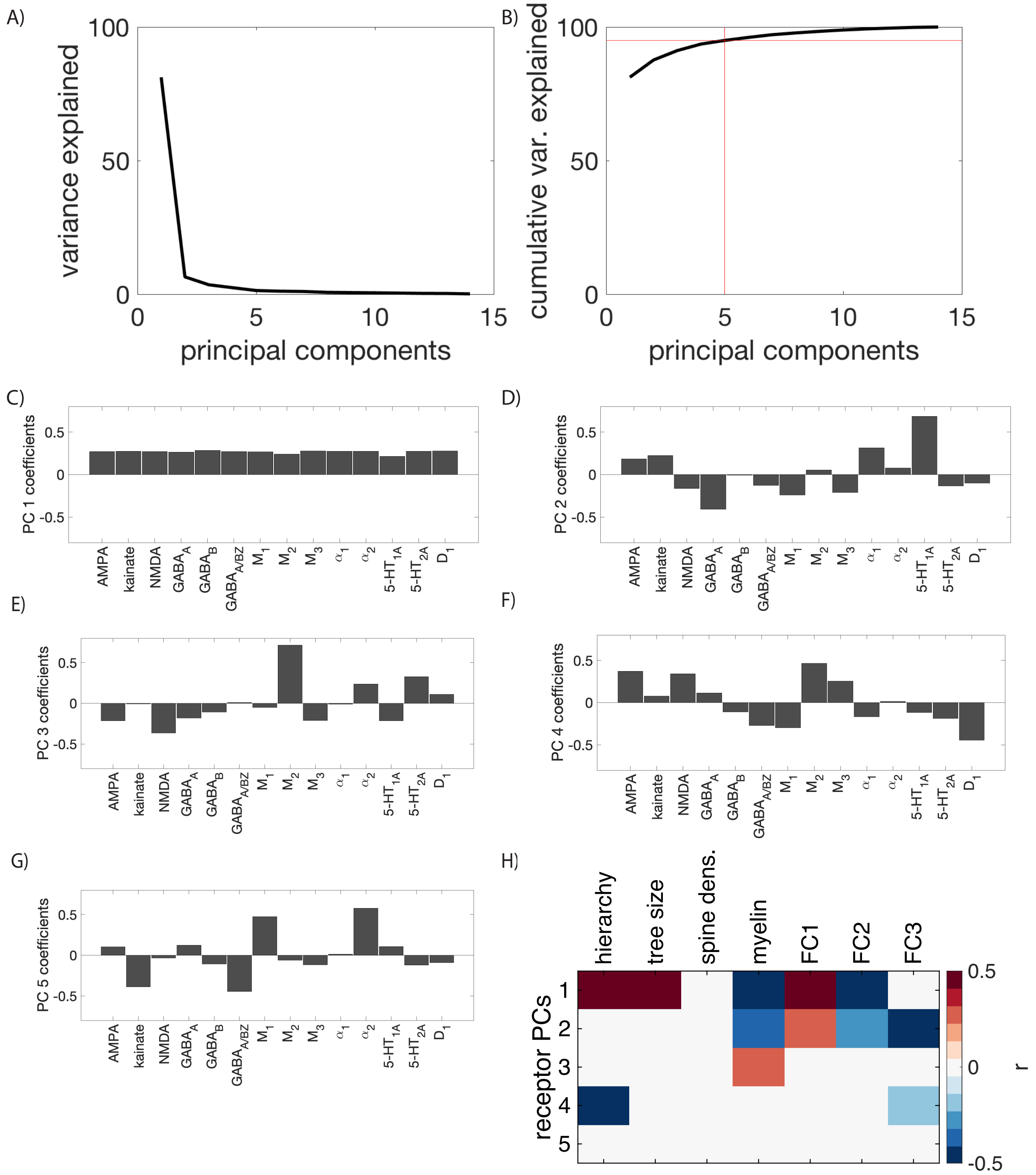

Figure S7. The spatial pattern of receptor gradients 2-5 depends on individual receptor types. The gradient dependence assesses how much the spatial pattern of a gradient changes if one receptor type is removed. The spatial pattern of PC1 is not strongly dependent on any one individual receptor, indicating that the principal receptor gradient captures strong underlying spatial trends across all receptor types. In contrast, the spatial patterns of PCs 2-5 are more dependent on individual receptor types.

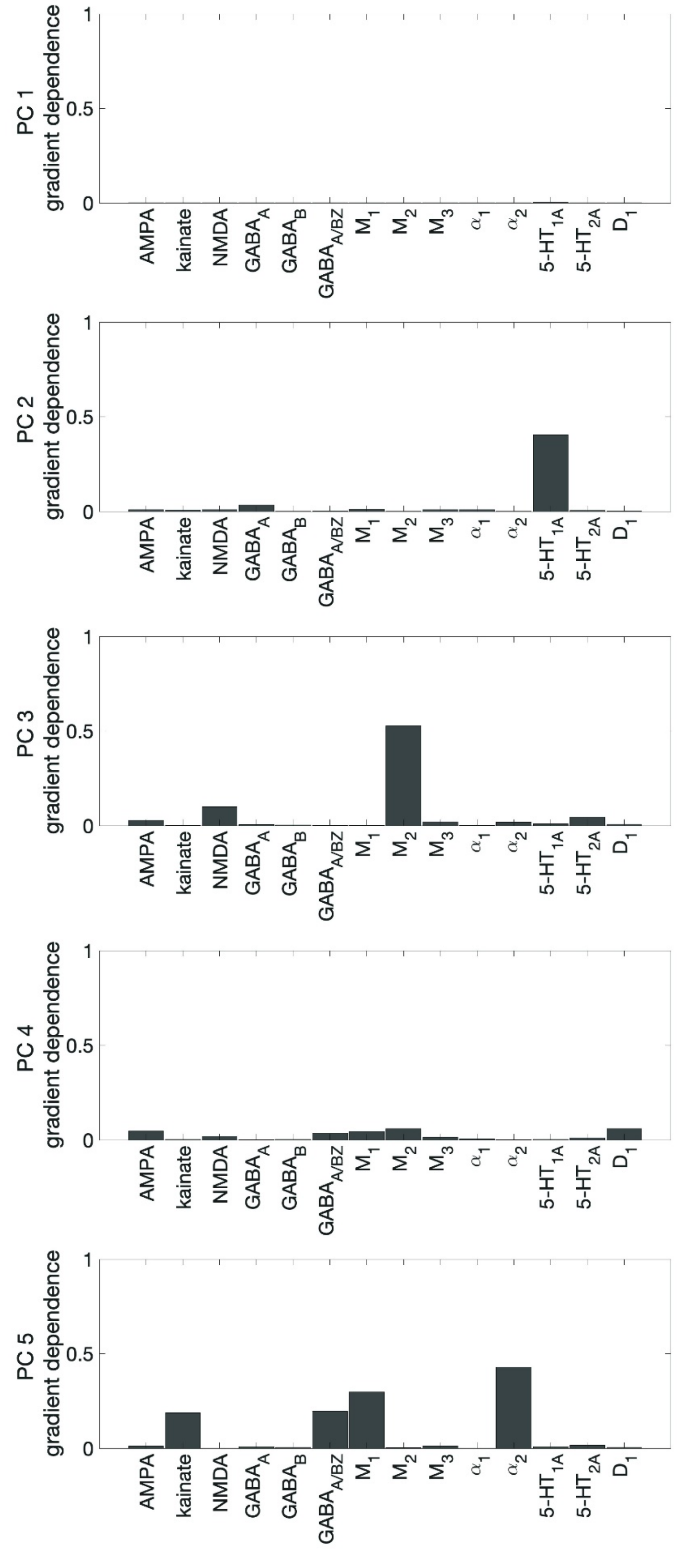

Figure S8. Principal components of raw receptor data correlate with gradients of anatomical and functional cortical organization. A) The Pearson's r for correlations between each principal component of the raw receptor data and each of the structural and functional maps of brain organization. Thresholded at p (two-sided) <0.05, uncorrected. FC = functional connectivity gradient. Note that different numbers of data points were available for different maps. B) Significant (uncorrected) relationships between receptor PC1 of the raw receptor data and gradients of i) dendritic tree size ($r(19) = 0.59$, $p = 0.005$), ii) T1w/T2w ratio (proposed myelin marker, ($r(107) = -0.80$, $p = 1x10^{-25}$) and iii) functional connectivity gradient 2 ($r(107) = -0.70$, $p = 2x10^{-17}$). C) Significant (uncorrected) relationships between receptor PC2 of the raw receptor data and i) the cortical hierarchy calculated via inter-areal connectivity patterns ($r(39)=0.80$, $p = 3x10^{-8}$), ii) dendritic tree size ($r(19) = 0.66$, $p = 0.001$), iii) peak spine density ($r(20) = 0.51$, $p = 0.0156$) and iv) functional connectivity gradient 1 (model: $X^2(107) = 46.5$, $r^2 = 0.44$, $p = 9x10^{-12}$, coefficient: estimate = 2.55 [SE 0.64], $t = 3.93$, $p = 8.5 \times 10^{-5}$).

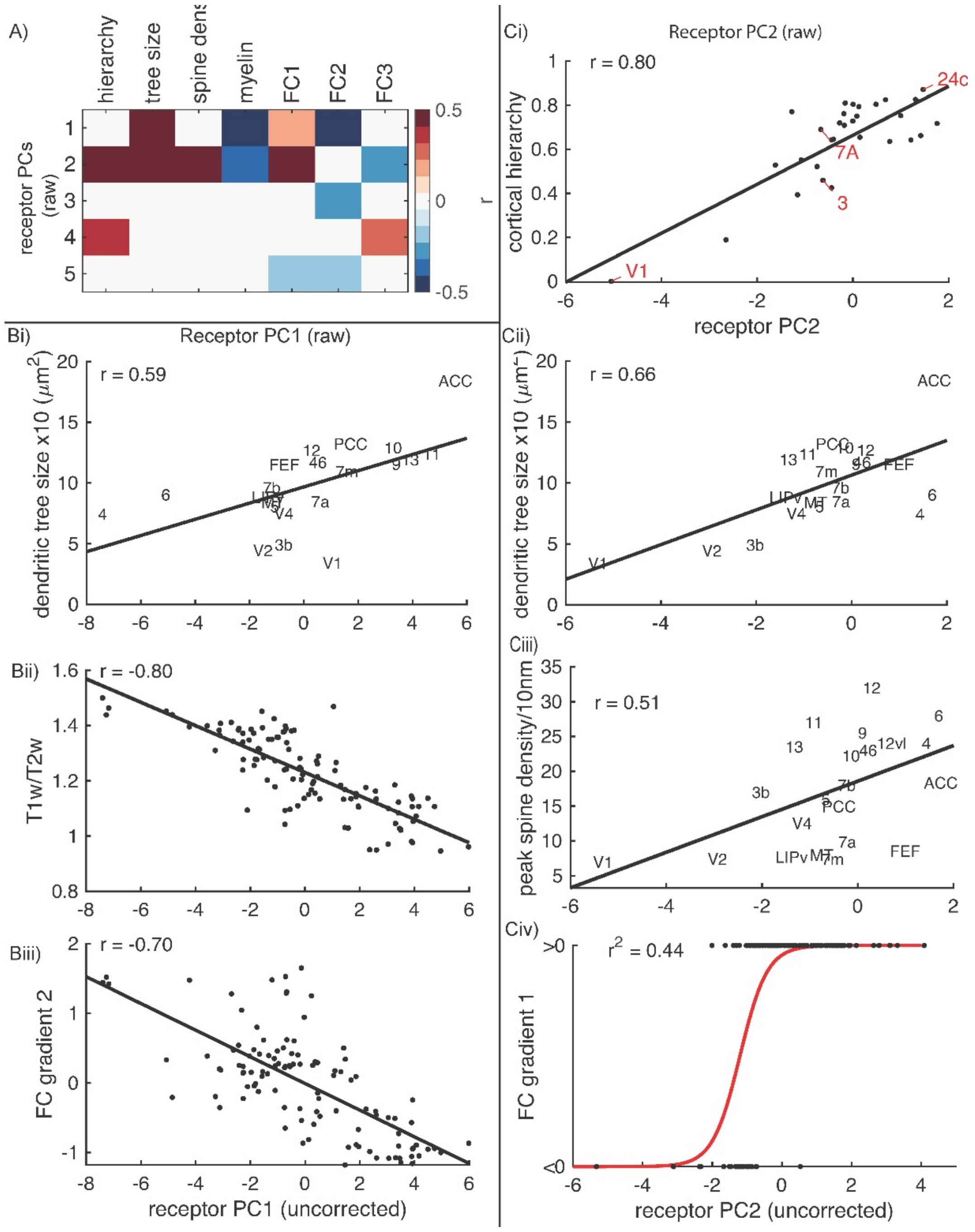

Figure S9. An inverse relationship between cortical myelin and receptor density in the primary motor and parietal association cortex. Receptor-, cyto-, and myeloarchitecture of the macaque primary motor cortex (area 4a) and of parietal association area PEipe. Changes in Grey Level Index, which represents a measure of the volume fraction of cell bodies, in myelin density and in receptor concentration (in fmol/mg protein) throughout the cortical depth are provided by the profile curve overlaid onto each section. Note that the scale has been optimized for each profile to provide the best visualization of changes in receptor densities throughout the cortical ribbon. Roman and Arabic numerals indicate cyto- and myeloarchitectonic layers, respectively. Positions of cytoarchitectonic layers were transferred to the neighboring receptor images.

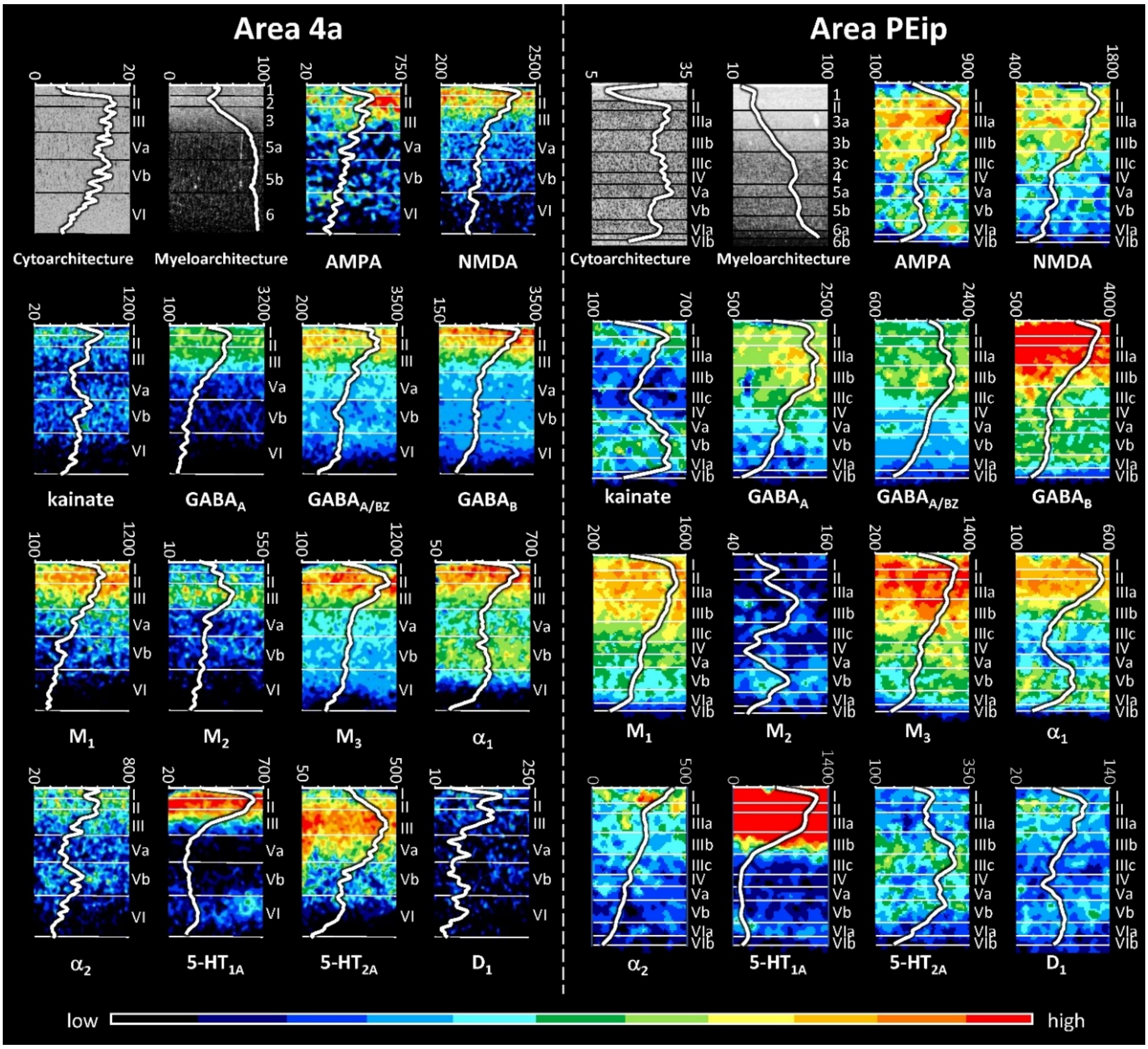

Figure S10. The serotonin 5-HT$_{1A}$ and 5-HT$_{2A}$ receptor density in higher cognitive networks. Top-left. The Yeo, Krienen et al. cognitive networks in macaque cortex. Right. The mean receptor density for 5-HT$_{1A}$ receptors and 5-HT$_{2A}$ receptors in the higher cognitive networks. Note, both receptor types were weakly expressed (per neuron) in the sensory networks. Bottom-left. There was no significant correlation between 5-HT$_{2A}$ expression and receptor PC2.

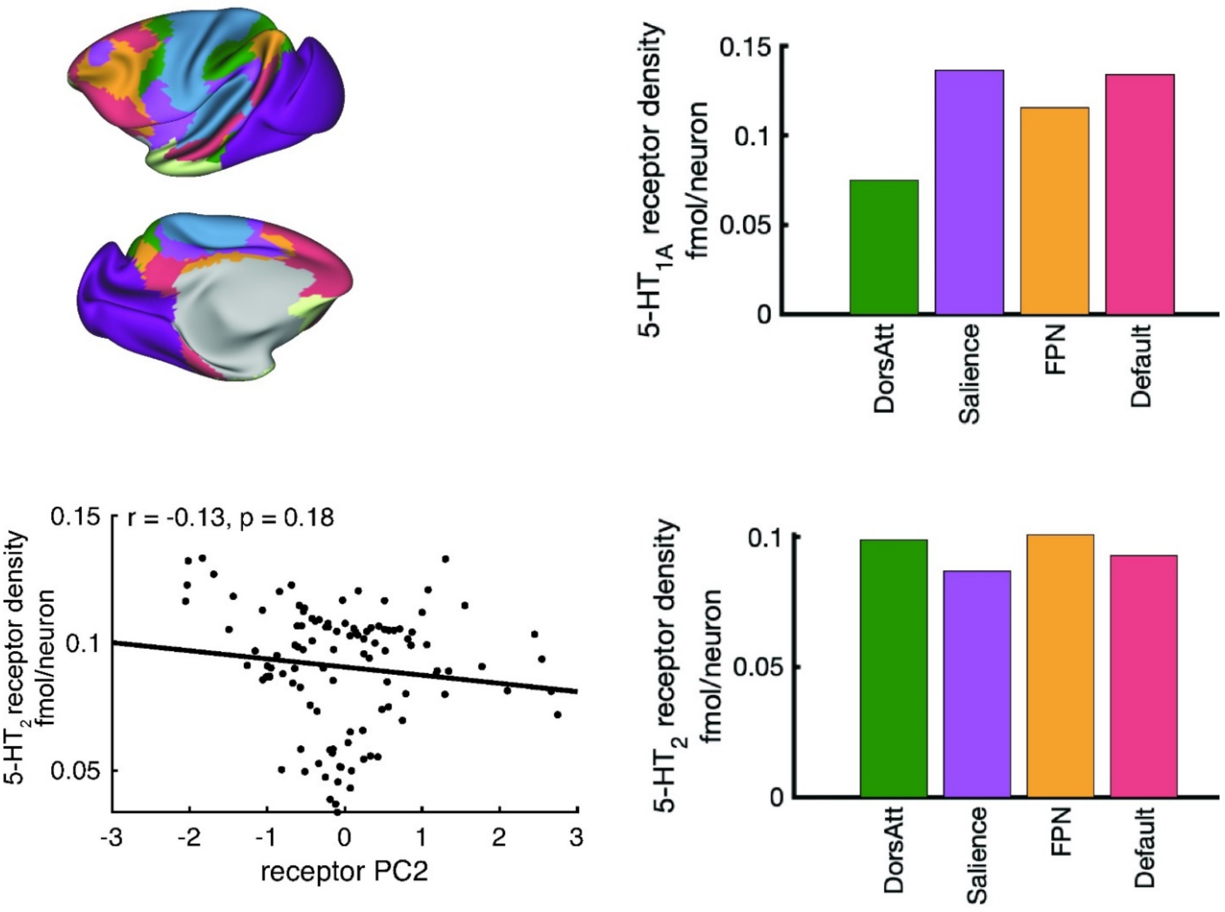

Figure S11. Variable relationships between gene and receptor expression across receptors. Human gene expression mapped onto the macaque cortex was correlated with macaque receptor expression, for 41 genes corresponding to 14 receptors. Pearson’s r correlations, two-sided p-values, uncorrected for multiple comparisons. Exact p-values are shown on right. Genes are coloured according to their corresponding neurotransmitter and receptor. Correlations that pass the spatial correction are shown in a red star. Correlations that pass a less stringent threshold ($p<0.005$, uncorrected) are shown in a blue star. Non-significant correlations are shown in black. We additionally performed Pearson’s r correlations of two measures (T1w/T2w ratio – purple line and cortical thickness – purple dashed line) that were acquired in the similar ways in both species. This gives an estimate of the maximum correlation to be expected for cross-species comparisons. The lower bound of the 95% confidence interval of the human-macaque cortical thickness correlation is additionally shown.

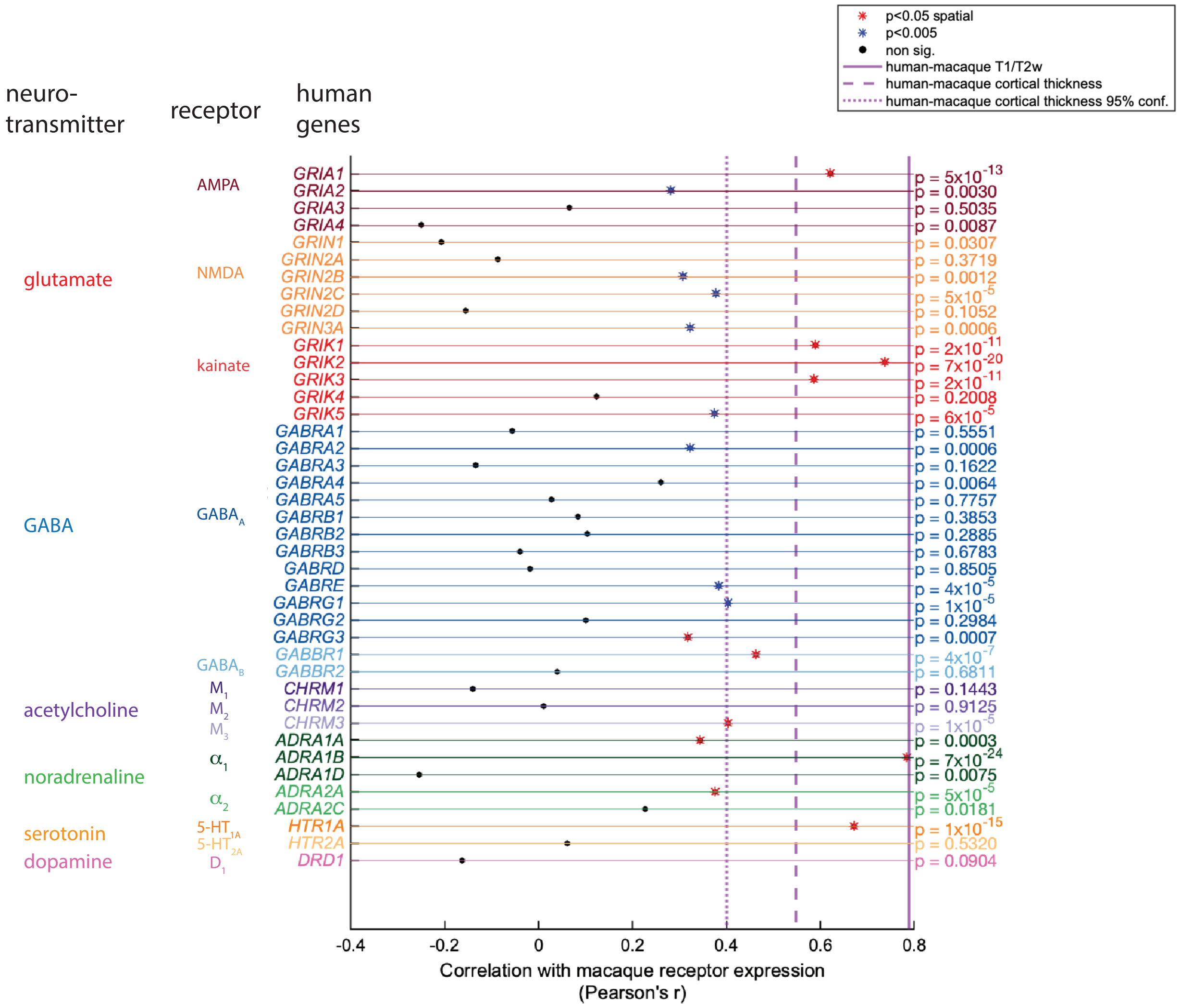

Supplementary Table 1. Mean receptor density per neuron (fmol/neuron).

	**AMPA**	**kainate**	**NMDA**	**$GABA_A$**	**$GABA_B$**	**$GABA_{A/BZ}$**	**M_1**	**M_2**	**M_3**	**α_1**	**α_2**	**$5HT_{1A}$**	**$5HT_{2A}$**	**D_1**
**10d**	0.1424	0.2609	0.3967	0.4683	0.5365	0.6476	0.295	0.0415	0.2521	0.1554	0.0963	0.1705	0.1032	0.0276
**10md**	0.1503	0.2572	0.4248	0.4734	0.5794	0.667	0.2916	0.0452	0.245	0.1398	0.0969	0.1805	0.1057	0.027
**10mv**	0.1611	0.2595	0.4621	0.4888	0.6374	0.6897	0.2816	0.0429	0.2563	0.1304	0.0955	0.2029	0.0958	0.0253
**10o**	0.1523	0.2609	0.4391	0.5064	0.6314	0.6843	0.2955	0.0449	0.268	0.1373	0.104	0.2009	0.1043	0.0247
**11l**	0.1793	0.2361	0.4604	0.5579	0.786	0.603	0.2929	0.0496	0.2788	0.136	0.1058	0.1529	0.1067	0.029
**11m**	0.1724	0.2201	0.4652	0.5149	0.7178	0.5714	0.3065	0.0497	0.2846	0.1393	0.0996	0.1521	0.1067	0.0277
**12l**	0.1785	0.2486	0.4103	0.429	0.6239	0.5675	0.2114	0.0457	0.2346	0.1458	0.0936	0.1583	0.1055	0.0201
**12m**	0.1807	0.2363	0.4656	0.528	0.6554	0.5506	0.3046	0.065	0.2811	0.143	0.1163	0.1389	0.1124	0.0294
**12o**	0.2057	0.2514	0.4713	0.4946	0.6828	0.6882	0.2432	0.0659	0.2642	0.1442	0.1244	0.1664	0.1206	0.0278
**12r**	0.1733	0.2436	0.4029	0.5198	0.7237	0.573	0.2939	0.0558	0.2711	0.1238	0.0912	0.153	0.1087	0.0246
**13b**	0.156	0.2336	0.4482	0.4666	0.6761	0.5377	0.2887	0.0506	0.2651	0.1373	0.1026	0.1606	0.1046	0.0276
**13l**	0.2257	0.2358	0.4864	0.541	0.6367	0.6545	0.2745	0.0857	0.2689	0.135	0.1253	0.1099	0.1138	0.0207
**13m**	0.2343	0.2802	0.4612	0.505	0.5289	0.5041	0.2837	0.0845	0.3031	0.1427	0.1266	0.1204	0.1168	0.0221
**14r**	0.1458	0.233	0.3831	0.4163	0.717	0.4703	0.266	0.0429	0.2296	0.1353	0.0793	0.154	0.0941	0.0259
**44**	0.1418	0.1586	0.3236	0.3859	0.5456	0.4576	0.238	0.0415	0.1891	0.1238	0.1073	0.1618	0.1054	0.0246
**45A**	0.1371	0.1986	0.3071	0.3945	0.5073	0.4888	0.2179	0.0465	0.2004	0.1048	0.0842	0.094	0.0974	0.0182
**45B**	0.1439	0.153	0.3247	0.3629	0.4713	0.4798	0.2391	0.0382	0.1808	0.114	0.0997	0.1209	0.1029	0.0236
**4a**	0.0596	0.0578	0.1852	0.1545	0.2208	0.3178	0.0966	0.0168	0.1114	0.0696	0.0279	0.0384	0.0544	0.0097
**4m**	0.0878	0.0852	0.2429	0.209	0.285	0.4199	0.1096	0.0231	0.1404	0.0944	0.0371	0.0565	0.0657	0.0139
**4p**	0.0571	0.0504	0.1643	0.143	0.2478	0.294	0.091	0.0164	0.1066	0.066	0.0328	0.0403	0.0557	0.0099
**8Ad**	0.1236	0.1876	0.261	0.2993	0.5294	0.4557	0.2198	0.0449	0.1812	0.1178	0.074	0.1139	0.097	0.0211
**8Av**	0.1076	0.1562	0.237	0.296	0.4368	0.4692	0.1669	0.0422	0.1669	0.0848	0.0688	0.0646	0.0853	0.021
**8Bd**	0.1294	0.198	0.2571	0.347	0.5461	0.6155	0.2315	0.0572	0.1977	0.13	0.0856	0.1308	0.1067	0.023
**8Bm**	0.162	0.2574	0.2933	0.3738	0.5988	0.5739	0.2225	0.0563	0.2077	0.1419	0.0919	0.1561	0.112	0.0239
**8Bs**	0.1267	0.1567	0.2585	0.3467	0.5342	0.524	0.2155	0.0452	0.1882	0.1168	0.0898	0.1339	0.1	0.0222
**9d**	0.1468	0.212	0.3604	0.4507	0.6315	0.56	0.2778	0.0489	0.2332	0.1438	0.103	0.1751	0.106	0.0261
**9m**	0.1484	0.2235	0.3149	0.3905	0.5464	0.4774	0.2384	0.0442	0.2038	0.1351	0.086	0.1681	0.0991	0.0242
**A46d**	0.1577	0.1913	0.3899	0.4763	0.5993	0.5041	0.3058	0.054	0.2435	0.1395	0.112	0.1287	0.1097	0.0264
**A46dr**	0.1472	0.235	0.349	0.4595	0.5706	0.5112	0.2822	0.0566	0.2416	0.1316	0.0952	0.1455	0.1077	0.0253
**A46v**	0.1748	0.1947	0.4169	0.505	0.6871	0.5189	0.3177	0.0554	0.26	0.1392	0.1162	0.1341	0.1148	0.0276

**A46vr**	0.152	0.231	0.372	0.4612	0.5908	0.5283	0.2805	0.0546	0.2509	0.1222	0.0932	0.137	0.1063	0.0244
**F2d**	0.0904	0.1649	0.2027	0.2295	0.4693	0.4433	0.2155	0.0435	0.1433	0.1258	0.089	0.1489	0.089	0.0219
**F2v**	0.0785	0.1309	0.1532	0.2095	0.3283	0.3253	0.184	0.0389	0.1216	0.102	0.0647	0.0895	0.0801	0.0198
**F3**	0.1265	0.2379	0.3007	0.297	0.5869	0.5215	0.2735	0.0631	0.1893	0.1641	0.1117	0.1899	0.1148	0.0285
**F4d**	0.0803	0.099	0.1655	0.2027	0.3446	0.3827	0.1312	0.0233	0.1102	0.0901	0.0602	0.0637	0.074	0.0168
**F4s**	0.0923	0.1144	0.1965	0.2378	0.4108	0.4404	0.1231	0.0359	0.1328	0.1017	0.0634	0.0864	0.0847	0.0216
**F4v**	0.0976	0.1439	0.2219	0.2932	0.4554	0.4197	0.1624	0.0293	0.1256	0.1091	0.0709	0.0917	0.075	0.0189
**F5d**	0.2144	0.1885	0.3181	0.3306	0.5698	0.5472	0.1945	0.0403	0.1782	0.1289	0.096	0.1407	0.0993	0.0223
**F5s**	0.1559	0.1839	0.3257	0.3578	0.5755	0.4991	0.2121	0.0404	0.1958	0.1299	0.1005	0.1421	0.105	0.0227
**F5v**	0.2506	0.26	0.4874	0.4553	0.8208	0.6992	0.2844	0.0563	0.2466	0.1688	0.1407	0.2047	0.1209	0.0251
**F6**	0.1591	0.2614	0.3492	0.3868	0.6648	0.5393	0.3112	0.0651	0.2035	0.1732	0.1153	0.2042	0.133	0.0286
**F7d**	0.1043	0.1843	0.2232	0.3133	0.5482	0.584	0.2565	0.0636	0.18	0.1358	0.0886	0.1327	0.1167	0.0241
**F7i**	0.0979	0.1685	0.2132	0.2525	0.4339	0.4233	0.206	0.0459	0.1564	0.1208	0.0838	0.1177	0.1016	0.023
**F7s**	0.1174	0.163	0.2456	0.2936	0.4534	0.4218	0.2316	0.0474	0.1662	0.1211	0.0881	0.122	0.105	0.0244
**P46d**	0.1549	0.1922	0.3438	0.4502	0.5685	0.5546	0.2779	0.0502	0.2307	0.1227	0.1077	0.132	0.1063	0.0221
**P46dr**	0.1481	0.2242	0.3048	0.3829	0.5429	0.51	0.2418	0.053	0.2248	0.1196	0.0825	0.1316	0.1046	0.0223
**P46v**	0.1611	0.189	0.3667	0.4823	0.6785	0.6379	0.2681	0.0467	0.2443	0.1293	0.1117	0.1429	0.1092	0.0238
**P46vr**	0.1423	0.2126	0.3112	0.3807	0.5537	0.5244	0.173	0.0468	0.2257	0.1102	0.0892	0.1215	0.1016	0.0227
**1**	0.0798	0.0941	0.1936	0.2564	0.2926	0.2803	0.1487	0.0287	0.1269	0.067	0.0515	0.04	0.0515	0.0131
**2**	0.0894	0.0934	0.1957	0.2695	0.3248	0.3112	0.1578	0.0258	0.1424	0.0701	0.0509	0.0491	0.0568	0.0154
**3al**	0.0877	0.0832	0.2179	0.29	0.3763	0.2975	0.1667	0.0333	0.1522	0.0892	0.0534	0.0773	0.0651	0.0164
**3am**	0.086	0.0854	0.1985	0.2654	0.3302	0.2779	0.1315	0.0267	0.1309	0.0729	0.0434	0.0535	0.0499	0.0141
**3bl**	0.0728	0.0674	0.2126	0.2477	0.2701	0.2506	0.1521	0.0466	0.13	0.062	0.0543	0.0363	0.0581	0.0116
**3bm**	0.0818	0.0936	0.2178	0.2851	0.3474	0.2868	0.1579	0.0354	0.1455	0.071	0.0504	0.0554	0.0584	0.0146
**AIP**	0.1456	0.1526	0.3622	0.4372	0.5385	0.4415	0.2606	0.0361	0.231	0.1154	0.0863	0.0984	0.09	0.0217
**DP**	0.1121	0.1749	0.2917	0.3736	0.4456	0.5317	0.2565	0.0379	0.199	0.0917	0.0689	0.0543	0.0866	0.0225
**LIPd**	0.1772	0.2167	0.5106	0.5924	0.7353	0.7086	0.3757	0.0501	0.2884	0.1367	0.1067	0.118	0.1334	0.03
**LIPv**	0.1619	0.2014	0.4535	0.5515	0.6516	0.6271	0.3333	0.0523	0.2639	0.1137	0.0994	0.0764	0.1323	0.0285
**LOP**	0.1323	0.2094	0.432	0.5102	0.5866	0.6483	0.3234	0.0512	0.2584	0.1159	0.1082	0.0523	0.1228	0.0278
**MIPd**	0.1293	0.1527	0.3291	0.4342	0.5489	0.4791	0.2587	0.0342	0.2068	0.1103	0.0788	0.0834	0.0951	0.0234
**MIPv**	0.1184	0.1407	0.3195	0.4216	0.5106	0.5247	0.246	0.0359	0.1968	0.0934	0.0784	0.067	0.0969	0.0215
**Opt**	0.1906	0.263	0.5161	0.6066	0.7483	0.786	0.3634	0.0559	0.302	0.1516	0.1055	0.1103	0.1271	0.0318
**PEc**	0.1394	0.1878	0.3511	0.4511	0.5804	0.6301	0.2739	0.0418	0.2342	0.1242	0.0712	0.0853	0.0902	0.0247

**PEci**	0.137	0.1665	0.3719	0.4009	0.559	0.4781	0.2696	0.033	0.2044	0.1159	0.0782	0.1226	0.0901	0.023
**PEipe**	0.1336	0.1348	0.3078	0.4017	0.5283	0.4588	0.2415	0.0343	0.196	0.0998	0.0714	0.0911	0.0826	0.021
**PEipi**	0.1413	0.1451	0.348	0.453	0.5922	0.527	0.2729	0.0336	0.234	0.1138	0.0842	0.0912	0.091	0.0231
**PEl**	0.1264	0.1446	0.3234	0.3753	0.503	0.4859	0.231	0.0329	0.1837	0.0976	0.0693	0.0753	0.0842	0.0223
**PEla**	0.1283	0.146	0.3664	0.3922	0.538	0.5574	0.2424	0.0369	0.1877	0.106	0.0669	0.0826	0.0879	0.0239
**PF**	0.1077	0.1272	0.2918	0.3473	0.4247	0.387	0.2004	0.0308	0.1792	0.0922	0.0712	0.0656	0.0757	0.017
**PFG**	0.1984	0.2405	0.4729	0.5555	0.6655	0.5928	0.3432	0.0526	0.2993	0.1571	0.1078	0.1067	0.1129	0.0274
**PFop**	0.1619	0.1924	0.3949	0.4551	0.5411	0.4698	0.2935	0.0478	0.247	0.1276	0.0972	0.1004	0.0985	0.0222
**PG**	0.1955	0.258	0.5021	0.5328	0.7554	0.6776	0.3304	0.0464	0.2894	0.1564	0.1093	0.1308	0.1202	0.0303
**PGm**	0.1533	0.1941	0.3566	0.4405	0.5828	0.5981	0.2614	0.0405	0.222	0.119	0.0759	0.1011	0.0992	0.0244
**PGop**	0.1897	0.2544	0.4981	0.5445	0.7565	0.6209	0.3428	0.0424	0.2818	0.1626	0.1239	0.1385	0.1228	0.0281
**PIP**	0.121	0.1716	0.4301	0.4856	0.5899	0.5649	0.2974	0.0496	0.2308	0.1012	0.1078	0.0385	0.1165	0.0253
**PPt**	0.158	0.205	0.4655	0.516	0.6302	0.6416	0.3297	0.04	0.2535	0.1276	0.1041	0.0993	0.1184	0.027
**TSA**	0.1176	0.1404	0.2826	0.3751	0.4911	0.3896	0.2209	0.0307	0.1992	0.103	0.0787	0.0814	0.0733	0.0204
**V6Adl**	0.1209	0.1516	0.3087	0.4008	0.4785	0.4614	0.2448	0.0351	0.1975	0.0916	0.0696	0.0658	0.087	0.0226
**V6Adm**	0.108	0.156	0.3129	0.3958	0.4978	0.5018	0.2573	0.0381	0.1976	0.098	0.0618	0.0626	0.0856	0.0227
**V6Avl**	0.1012	0.1307	0.303	0.3771	0.4318	0.4177	0.2319	0.0371	0.1913	0.0825	0.0774	0.0371	0.0912	0.0206
**V6Avm**	0.0896	0.1405	0.3091	0.3576	0.4861	0.4103	0.2235	0.0437	0.1871	0.0827	0.062	0.0562	0.0867	0.022
**V6l**	0.0589	0.086	0.2009	0.2409	0.2937	0.2834	0.1558	0.0266	0.1259	0.0498	0.052	0.0198	0.0584	0.0135
**V6m**	0.052	0.0857	0.1916	0.2264	0.2615	0.2424	0.1331	0.0272	0.1142	0.0476	0.0434	0.0215	0.0527	0.0123
**VIP**	0.1369	0.1469	0.3685	0.4958	0.5863	0.5431	0.277	0.0387	0.2355	0.1098	0.0879	0.0776	0.1053	0.0251
**V1**	0.0256	0.0424	0.117	0.165	0.1634	0.1953	0.0981	0.0315	0.0736	0.0212	0.0408	0.0062	0.0368	0.006
**V2d**	0.0394	0.0738	0.1461	0.1944	0.2172	0.2411	0.1131	0.0211	0.0847	0.0326	0.0361	0.0123	0.0386	0.0084
**V2v**	0.0289	0.0538	0.1233	0.1586	0.167	0.2026	0.107	0.0178	0.0703	0.0229	0.0317	0.0103	0.0336	0.0068
**V3A**	0.0537	0.0699	0.1687	0.2068	0.2349	0.2341	0.1249	0.0189	0.1055	0.0418	0.0415	0.0206	0.0473	0.0105
**V3d**	0.0476	0.0677	0.1817	0.2211	0.2462	0.2437	0.1358	0.0268	0.109	0.0374	0.0442	0.0132	0.0495	0.0112
**V3v**	0.0301	0.1039	0.1234	0.162	0.2112	0.2563	0.0869	0.0191	0.0894	0.033	0.0375	0.0139	0.043	0.0092
**V4dl**	0.0662	0.0956	0.1707	0.2047	0.2362	0.2588	0.1384	0.0197	0.0981	0.0466	0.0385	0.0227	0.0455	0.0111
**V4dm**	0.0822	0.1136	0.2404	0.3142	0.31	0.3398	0.1833	0.0133	0.1391	0.0546	0.0541	0.0251	0.0503	0.0139
**V4v**	0.0346	0.1164	0.1523	0.1744	0.2565	0.3069	0.1005	0.0218	0.1087	0.0418	0.0428	0.0185	0.0513	0.0111
**23c**	0.1415	0.1629	0.4016	0.3069	0.4326	0.3947	0.1527	0.0428	0.2293	0.1285	0.0814	0.1784	0.0799	0.0188
**23d**	0.1505	0.1868	0.4338	0.3556	0.4679	0.4407	0.1804	0.0369	0.2188	0.1144	0.0534	0.1604	0.0696	0.0175
**24ab**	0.2166	0.254	0.3764	0.3629	0.6288	0.5772	0.3393	0.0432	0.1927	0.159	0.1053	0.3014	0.0937	0.0237

**24c**	0.2021	0.2276	0.4057	0.4034	0.702	0.6669	0.3188	0.0557	0.2116	0.1595	0.1156	0.2042	0.089	0.0235
**24d**	0.1648	0.1529	0.3731	0.3312	0.4583	0.4153	0.2224	0.0364	0.2123	0.1277	0.0756	0.1906	0.0878	0.0167
**25**	0.2047	0.2949	0.4187	0.2667	0.7594	0.4242	0.1989	0.0447	0.2474	0.1556	0.0662	0.2449	0.0811	0.0243
**29/30**	0.1403	0.1274	0.2675	0.3421	0.44	0.4167	0.1706	0.0337	0.1842	0.0672	0.0632	0.0667	0.0974	0.0161
**31**	0.1519	0.1747	0.3378	0.4319	0.6351	0.4289	0.3041	0.0345	0.245	0.1321	0.0757	0.1386	0.1077	0.0203
**a24'ab**	0.1796	0.2444	0.2957	0.3498	0.5752	0.5249	0.3092	0.0333	0.2033	0.154	0.1184	0.3011	0.0719	0.0229
**a24'c**	0.1723	0.2242	0.3234	0.4135	0.6459	0.5509	0.2977	0.0539	0.1941	0.1525	0.1332	0.2435	0.0908	0.0251
**d23ab**	0.1681	0.1642	0.3369	0.3929	0.5589	0.4247	0.2966	0.0412	0.2175	0.1309	0.0675	0.0881	0.1008	0.0204
**p24'ab**	0.1738	0.1773	0.3676	0.3265	0.3628	0.3765	0.1748	0.0221	0.1928	0.1319	0.0724	0.236	0.0813	0.0163
**p32**	0.2185	0.2353	0.4962	0.4092	0.6687	0.495	0.2117	0.0477	0.2444	0.1648	0.1358	0.15	0.1043	0.0273
**s32**	0.2092	0.2586	0.4024	0.3799	0.6505	0.526	0.2114	0.0605	0.2157	0.1526	0.0872	0.2742	0.1034	0.0233
**v23ab**	0.0738	0.1262	0.194	0.2518	0.3913	0.314	0.1001	0.0224	0.1452	0.0783	0.0436	0.0662	0.0553	0.0123
**MT**	0.0582	0.1363	0.1601	0.2132	0.2697	0.2645	0.1262	0.025	0.1091	0.0521	0.0512	0.0262	0.0609	0.0124

Supplementary Table 2. Standard deviation of receptor density per neuron (fmol/neuron).

	AMPA	kainate	NMDA	$GABA_A$	$GABA_B$	$GABA_{A/BZ}$	M_1	M_2	M_3	α_1	α_2	$5HT_{1A}$	$5HT_{2A}$	D_1
**10d**	0.0034	0.0122	0.1046	0.0526	0.1123	0.3322	0.1074	0.0145	0.0332	0.0259	0.0156	0.0409	0.0236	0.0077
**10md**	0.0162	0.0145	0.0367	0.0227	0.1270	0.2708	0.0961	0.0134	0.0296	0.0156	0.0117	0.0389	0.0156	0.0074
**10mv**	0.0358	0.0136	0.0472	0.0469	0.1699	0.2782	0.1145	0.0117	0.0227	0.0355	0.0131	0.0702	0.0205	0.0060
**10o**	0.0278	0.0099	0.0136	0.0165	0.1805	0.2654	0.1148	0.0134	0.0242	0.0256	0.0119	0.0614	0.0159	0.0034
**11l**	0.0227	0.0427	0.0244	0.0422	0.1486	0.0536	0.0826	0.0172	0.0186	0.0123	0.0092	0.0393	0.0161	0.0100
**11m**	0.0200	0.0168	0.0391	0.0223	0.1133	0.0551	0.0731	0.0211	0.0420	0.0106	0.0103	0.0608	0.0188	0.0091
**12l**	0.0335	0.0193	0.0054	0.0712	0.0403	0.1146	0.1226	0.0247	0.0153	0.0241	0.0113	0.0488	0.0133	0.0017
**12m**	0.0212	0.0062	0.0436	0.0739	0.0536	0.1227	0.0898	0.0197	0.0109	0.0135	0.0135	0.0177	0.0129	0.0097
**12o**	0.0573	0.0098	0.0225	0.1117	0.0260	0.0310	0.0260	0.0242	0.0106	0.0109	0.0095	0.0325	0.0121	0.0071
**12r**	0.0383	0.0420	0.0126	0.0849	0.0732	0.0452	0.1015	0.0254	0.0212	0.0100	0.0109	0.0320	0.0114	0.0023
**13b**	0.0290	0.0355	0.0691	0.0330	0.0966	0.1199	0.0921	0.0168	0.0142	0.0239	0.0236	0.0719	0.0159	0.0054
**13l**	N/A	N/A	N/A	N/A	N/A	N/A	N/A	N/A	N/A	N/A	N/A	N/A	N/A	N/A
**13m**	N/A	N/A	N/A	N/A	N/A	N/A	N/A	N/A	N/A	N/A	N/A	N/A	N/A	N/A
**14r**	0.0546	0.0296	0.1080	0.0509	0.0637	0.1034	0.1307	0.0151	0.0290	0.0375	0.0344	0.0455	0.0068	0.0023
**44**	0.0324	0.0185	0.0527	0.0353	0.0514	0.0567	0.0992	0.0053	0.0511	0.0249	0.0198	0.0591	0.0179	0.0088
**45A**	0.0108	0.0092	0.0127	0.0798	0.0552	0.1291	0.0607	0.0169	0.0042	0.0187	0.0079	0.0285	0.0127	0.0053
**45B**	0.0345	0.0111	0.0628	0.0419	0.0824	0.0546	0.0930	0.0037	0.0382	0.0191	0.0244	0.0353	0.0156	0.0101
**4a**	0.0138	0.0148	0.0508	0.0352	0.0036	0.1229	0.0174	0.0142	0.0103	0.0034	0.0105	0.0101	0.0113	0.0026
**4m**	0.0158	0.0226	0.0634	0.0389	0.0350	0.1528	0.0352	0.0218	0.0105	0.0042	0.0142	0.0066	0.0126	0.0037
**4p**	0.0160	0.0150	0.0450	0.0468	0.0073	0.0853	0.0344	0.0118	0.0030	0.0036	0.0103	0.0049	0.0105	0.0014
**8Ad**	0.0148	0.0153	0.0779	0.0214	0.0240	0.0859	0.0713	0.0095	0.0478	0.0180	0.0045	0.0127	0.0108	0.0100
**8Av**	0.0175	0.0332	0.0175	0.0462	0.0667	0.1246	0.0685	0.0072	0.0638	0.0321	0.0178	0.0329	0.0218	0.0082
**8Bd**	0.0233	0.0119	0.1013	0.0438	0.0310	0.0811	0.0466	0.0148	0.0293	0.0245	0.0119	0.0208	0.0111	0.0102
**8Bm**	0.0431	0.0091	0.1290	0.0972	0.1016	0.0733	0.0582	0.0138	0.0340	0.0293	0.0101	0.0258	0.0094	0.0110
**8Bs**	0.0150	0.0083	0.0978	0.0615	0.1246	0.0305	0.0217	0.0110	0.0262	0.0206	0.0048	0.0356	0.0128	0.0075
**9d**	0.0294	0.0141	0.0693	0.1095	0.0658	0.1468	0.0644	0.0109	0.0103	0.0101	0.0125	0.0212	0.0196	0.0082
**9m**	0.0160	0.0168	0.0730	0.1183	0.0589	0.1223	0.0765	0.0101	0.0077	0.0107	0.0080	0.0426	0.0139	0.0069
**A46d**	0.0185	0.0108	0.0091	0.0739	0.0253	0.1262	0.0551	0.0131	0.0105	0.0105	0.0165	0.0332	0.0182	0.0128
**A46dr**	0.0227	0.0250	0.0577	0.0753	0.0281	0.1140	0.0878	0.0094	0.0247	0.0233	0.0173	0.0298	0.0131	0.0108
**A46v**	0.0054	0.0247	0.0060	0.0963	0.0020	0.1157	0.0554	0.0134	0.0080	0.0105	0.0057	0.0278	0.0162	0.0111

**A46vr**	0.0156	0.0179	0.0406	0.0867	0.0563	0.1159	0.0781	0.0176	0.0097	0.0159	0.0045	0.0250	0.0082	0.0060
**F2d**	0.0125	0.0199	0.0479	0.0619	0.0192	0.0165	0.0533	0.0108	0.0354	0.0143	0.0106	0.0216	0.0184	0.0084
**F2v**	0.0100	0.0116	0.0351	0.0462	0.0100	0.0296	0.0503	0.0068	0.0278	0.0159	0.0114	0.0137	0.0198	0.0075
**F3**	0.0470	0.0141	0.1030	0.1141	0.0336	0.0859	0.0889	0.0117	0.0510	0.0228	0.0144	0.0238	0.0211	0.0134
**F4d**	0.0089	0.0096	0.0145	0.0311	0.0616	0.1004	0.0462	0.0056	0.0161	0.0040	0.0086	0.0016	0.0208	0.0056
**F4s**	0.0221	0.0046	0.0416	0.0278	0.0367	0.0831	0.0089	0.0103	0.0232	0.0246	0.0022	0.0197	0.0173	0.0078
**F4v**	0.0256	0.0480	0.0733	0.0798	0.0211	0.0528	0.0930	0.0107	0.0070	0.0004	0.0209	0.0026	0.0122	0.0046
**F5d**	0.0000	0.0119	0.0458	0.0378	0.0822	0.0742	0.1073	0.0160	0.0602	0.0116	0.0077	0.0403	0.0097	0.0063
**F5s**	0.0662	0.0061	0.0712	0.0194	0.1141	0.0831	0.1219	0.0091	0.0595	0.0216	0.0147	0.0388	0.0186	0.0078
**F5v**	0.0245	0.0168	0.0405	0.0670	0.1099	0.0670	0.1276	0.0322	0.0660	0.0114	0.0057	0.0482	0.0161	0.0027
**F6**	0.0372	0.0286	0.1092	0.0463	0.0242	0.0625	0.1099	0.0159	0.0766	0.0311	0.0148	0.0484	0.0300	0.0134
**F7d**	0.0250	0.0290	0.0923	0.0435	0.0417	0.0744	0.0664	0.0136	0.0531	0.0235	0.0130	0.0185	0.0235	0.0136
**F7i**	0.0290	0.0189	0.0689	0.0204	0.0456	0.0732	0.0700	0.0103	0.0557	0.0224	0.0098	0.0258	0.0253	0.0100
**F7s**	0.0181	0.0218	0.0829	0.0184	0.0649	0.0425	0.0815	0.0092	0.0514	0.0247	0.0158	0.0258	0.0215	0.0106
**P46d**	0.0215	0.0060	0.0785	0.0864	0.0308	0.0515	0.1003	0.0139	0.0044	0.0191	0.0098	0.0245	0.0082	0.0095
**P46dr**	0.0156	0.0039	0.0937	0.0772	0.0262	0.0722	0.0845	0.0156	0.0223	0.0192	0.0042	0.0092	0.0078	0.0109
**P46v**	0.0276	0.0149	0.0580	0.1028	0.0171	0.0459	0.0826	0.0127	0.0127	0.0196	0.0221	0.0326	0.0102	0.0091
**P46vr**	0.0089	0.0102	0.0227	0.0856	0.0357	0.0595	0.0111	0.0105	0.0172	0.0230	0.0141	0.0174	0.0114	0.0080
**1**	0.0166	0.0210	0.0278	0.0228	0.0314	0.0108	0.0188	0.0089	0.0190	0.0088	0.0060	0.0058	0.0099	0.0038
**2**	0.0066	0.0197	0.0158	0.0387	0.0541	0.0697	0.0260	0.0110	0.0222	0.0123	0.0067	0.0173	0.0102	0.0013
**3al**	0.0156	0.0135	0.0019	0.0345	0.0383	0.0000	0.0567	0.0146	0.0239	0.0029	0.0131	0.0214	0.0056	0.0004
**3am**	0.0133	0.0097	0.0172	0.0273	0.0246	0.0487	0.0392	0.0099	0.0099	0.0057	0.0085	0.0103	0.0109	0.0010
**3bl**	0.0108	0.0061	0.0290	0.0502	0.0360	0.0168	0.0401	0.0160	0.0159	0.0080	0.0013	0.0052	0.0111	0.0002
**3bm**	0.0109	0.0095	0.0265	0.0345	0.0258	0.0174	0.0382	0.0093	0.0080	0.0030	0.0128	0.0100	0.0122	0.0009
**AIP**	0.0214	0.0274	0.0220	0.0719	0.0801	0.0688	0.0513	0.0124	0.0276	0.0138	0.0164	0.0065	0.0231	0.0011
**DP**	0.0318	0.0106	0.0445	0.0623	0.0530	0.0278	0.0318	0.0127	0.0223	0.0087	0.0106	0.0117	0.0244	0.0021
**LIPd**	0.0241	0.0197	0.0764	0.0581	0.1122	0.1089	0.0771	0.0168	0.0384	0.0069	0.0095	0.0190	0.0307	0.0015
**LIPv**	0.0161	0.0088	0.0767	0.0577	0.0961	0.0534	0.0556	0.0153	0.0373	0.0095	0.0095	0.0062	0.0259	0.0011
**LOP**	0.0223	0.0216	0.0548	0.0607	0.0841	0.0724	0.0274	0.0132	0.0398	0.0194	0.0091	0.0238	0.0274	0.0022
**MIPd**	0.0174	0.0247	0.0508	0.0446	0.0823	0.0318	0.0266	0.0109	0.0228	0.0114	0.0136	0.0215	0.0201	0.0016
**MIPv**	0.0177	0.0158	0.0659	0.0493	0.0705	0.0498	0.0283	0.0103	0.0259	0.0073	0.0122	0.0169	0.0158	0.0016
**Opt**	0.0296	0.0373	0.0672	0.0782	0.1176	0.1165	0.0500	0.0179	0.0256	0.0128	0.0095	0.0157	0.0351	0.0037
**PEc**	0.0139	0.0174	0.0168	0.0312	0.0853	0.0168	0.0348	0.0130	0.0280	0.0062	0.0155	0.0269	0.0177	0.0033

**PEci**	0.0157	0.0244	0.0455	0.0796	0.1061	0.0260	0.0298	0.0143	0.0460	0.0089	0.0181	0.0149	0.0133	0.0005
**PEipe**	0.0033	0.0220	0.0637	0.0624	0.0704	0.0281	0.0248	0.0136	0.0205	0.0077	0.0154	0.0107	0.0217	0.0015
**PEipi**	0.0167	0.0307	0.0374	0.0541	0.0895	0.0360	0.0213	0.0126	0.0290	0.0117	0.0167	0.0091	0.0175	0.0015
**PEl**	0.0141	0.0329	0.0386	0.0484	0.1152	0.0660	0.0457	0.0139	0.0353	0.0155	0.0139	0.0155	0.0207	0.0038
**PEla**	0.0226	0.0196	0.0369	0.0497	0.0773	0.0839	0.0374	0.0157	0.0258	0.0077	0.0165	0.0104	0.0183	0.0021
**PF**	0.0071	0.0197	0.0195	0.0905	0.0695	0.0002	0.0308	0.0113	0.0246	0.0158	0.0182	0.0071	0.0170	0.0017
**PFG**	0.0146	0.0417	0.0515	0.1169	0.0837	0.0479	0.0556	0.0230	0.0428	0.0212	0.0172	0.0106	0.0278	0.0026
**PFop**	0.0278	0.0445	0.0530	0.0730	0.1086	0.0200	0.0232	0.0147	0.0370	0.0196	0.0209	0.0128	0.0203	0.0013
**PG**	0.0249	0.0351	0.0804	0.0910	0.1334	0.0566	0.0365	0.0183	0.0486	0.0168	0.0139	0.0117	0.0274	0.0015
**PGm**	0.0281	0.0172	0.0359	0.0512	0.0668	0.0029	0.0461	0.0110	0.0231	0.0075	0.0067	0.0281	0.0217	0.0032
**PGop**	0.0183	0.0292	0.1049	0.0651	0.1714	0.0859	0.0413	0.0132	0.0453	0.0197	0.0135	0.0150	0.0285	0.0022
**PIP**	0.0204	0.0211	0.0412	0.0565	0.1054	0.0433	0.0464	0.0146	0.0360	0.0225	0.0094	0.0191	0.0260	0.0028
**PPt**	0.0305	0.0187	0.0793	0.0704	0.1130	0.0362	0.0305	0.0060	0.0279	0.0095	0.0114	0.0225	0.0213	0.0019
**TSA**	0.0123	0.0251	0.0394	0.0433	0.0455	0.0128	0.0295	0.0118	0.0175	0.0047	0.0049	0.0155	0.0148	0.0012
**V6Adl**	0.0204	0.0255	0.0288	0.0538	0.0872	0.0057	0.0190	0.0122	0.0245	0.0109	0.0101	0.0114	0.0190	0.0022
**V6Adm**	0.0324	0.0181	0.0229	0.0686	0.0605	0.0135	0.0186	0.0108	0.0235	0.0086	0.0097	0.0165	0.0224	0.0030
**V6Avl**	0.0160	0.0049	0.0154	0.0547	0.0771	0.0363	0.0160	0.0100	0.0252	0.0076	0.0057	0.0106	0.0149	0.0011
**V6Avm**	0.0156	0.0201	0.0480	0.0930	0.0761	0.0000	0.0045	0.0167	0.0318	0.0159	0.0125	0.0085	0.0201	0.0011
**V6l**	0.0075	0.0060	0.0243	0.0492	0.0801	0.0000	0.0137	0.0092	0.0204	0.0101	0.0065	0.0088	0.0155	0.0013
**V6m**	0.0119	0.0124	0.0182	0.0421	0.0549	0.0138	0.0146	0.0094	0.0184	0.0099	0.0030	0.0078	0.0123	0.0007
**VIP**	0.0148	0.0325	0.0583	0.0503	0.1095	0.0168	0.0238	0.0151	0.0306	0.0161	0.0161	0.0071	0.0197	0.0019
**V1**	0.0045	0.0038	0.0131	0.0399	0.0345	0.0253	0.0186	0.0066	0.0148	0.0067	0.0034	0.0019	0.0094	0.0004
**V2d**	0.0094	0.0040	0.0153	0.0504	0.0182	0.0162	0.0335	0.0064	0.0119	0.0071	0.0017	0.0029	0.0116	0.0006
**V2v**	0.0032	0.0039	0.0061	0.0297	0.0236	0.0136	0.0031	0.0062	0.0077	0.0061	0.0051	0.0035	0.0088	0.0006
**V3A**	0.0050	0.0082	0.0050	0.0441	0.0452	0.0007	0.0193	0.0038	0.0159	0.0098	0.0075	0.0072	0.0115	0.0017
**V3d**	0.0079	0.0066	0.0141	0.0654	0.0442	0.0039	0.0159	0.0103	0.0120	0.0072	0.0045	0.0058	0.0124	0.0009
**V3v**	0.0052	0.0461	0.0477	0.0891	0.0078	0.0339	0.0515	0.0063	0.0094	0.0101	0.0052	0.0069	0.0132	0.0033
**V4dl**	0.0122	0.0164	0.0129	0.0438	0.0319	0.0270	0.0136	0.0066	0.0111	0.0051	0.0048	0.0048	0.0125	0.0008
**V4dm**	N/A	N/A	N/A	N/A	N/A	N/A	N/A	N/A	N/A	N/A	N/A	N/A	N/A	N/A
**V4v**	0.0090	0.0441	0.0395	0.0955	0.0284	0.0411	0.0658	0.0085	0.0069	0.0108	0.0111	0.0066	0.0142	0.0029
**23c**	0.0079	0.0056	0.0056	0.0211	0.0150	0.0023	0.0214	0.0043	0.0232	0.0135	0.0122	0.0112	0.0025	0.0041
**23d**	0.0236	0.0132	0.0262	0.0381	0.0158	0.0356	0.0000	0.0066	0.0132	0.0216	0.0015	0.0267	0.0071	0.0041
**24ab**	0.0282	0.0055	0.0737	0.0416	0.1105	0.0621	0.0582	0.0092	0.0321	0.0008	0.0084	0.0163	0.0161	0.0087

**24c**	0.0059	0.0072	0.0201	0.0196	0.0625	0.0617	0.0490	0.0008	0.0116	0.0052	0.0093	0.0181	0.0067	0.0101
**24d**	0.0387	0.0247	0.0401	0.0374	0.0470	0.1478	0.1040	0.0157	0.0133	0.0194	0.0234	0.0300	0.0141	0.0037
**25**	0.0132	0.0061	0.0684	0.0028	0.0632	0.0419	0.0188	0.0110	0.0207	0.0006	0.0094	0.0215	0.0234	0.0099
**29/30**	0.0067	0.0097	0.0293	0.0627	0.0265	0.0516	0.0146	0.0047	0.0139	0.0084	0.0146	0.0094	0.0161	0.0027
**31**	0.0073	0.0163	0.0087	0.0016	0.0138	0.0165	0.0304	0.0062	0.0217	0.0041	0.0035	0.0149	0.0155	0.0033
**a24'ab**	0.0262	0.0003	0.0480	0.0579	0.0528	0.0020	0.0457	0.0015	0.0130	0.0015	0.0061	0.0109	0.0084	0.0145
**a24'c**	0.0489	0.0408	0.1078	0.0628	0.0528	0.0944	0.0986	0.0151	0.0631	0.0257	0.0262	0.0740	0.0159	0.0145
**d23ab**	0.0255	0.0112	0.0435	0.0644	0.0512	0.0104	0.0311	0.0074	0.0196	0.0041	0.0120	0.0209	0.0171	0.0043
**p24'ab**	0.0231	0.0201	0.0483	0.0267	0.0361	0.0386	0.0013	0.0025	0.0267	0.0076	0.0086	0.0043	0.0150	0.0020
**p32**	0.0230	0.0134	0.0398	0.0298	0.0480	0.0017	0.0372	0.0080	0.0091	0.0028	0.0091	0.0318	0.0318	0.0074
**s32**	0.0193	0.0111	0.0142	0.0350	0.0486	0.0088	0.0347	0.0031	0.0230	0.0310	0.0040	0.0094	0.0151	0.0034
**v23ab**	0.0079	0.0114	0.0099	0.0459	0.0402	0.0582	0.0049	0.0037	0.0154	0.0029	0.0068	0.0110	0.0183	0.0023
**MT**	0.0058	0.0969	0.0601	0.0251	0.0267	0.0148	0.0383	0.0091	0.0041	0.0121	0.0086	0.0133	0.0121	0.0022

N/A: Note that due to artifacts of sectioning, receptor values were only available for one brain for areas V4dm, 13l and 13m, which prevented estimates of standard deviation.

Supplementary Table 3. Overlap of regions of the Julich Macaque Brain Atlas with the 7 cognitive networks of Yeo, Krienen et al in the macaque cortex. Overlap is expressed as a fraction.

	Visual	SomMot	DorsAtt	Salience	Limbic	FPN	Default
**10d**	0	0	0	0	0.34	0	0.66
**10md**	0	0	0	0	0	0	1
**10mv**	0	0	0	0	0.11	0	0.89
**10o**	0	0	0	0	0.28	0	0.72
**11l**	0	0	0	0	0.83	0.17	0
**11m**	0	0	0	0	0.9	0	0.1
**12l**	0	0	0	0	0	0	1
**12m**	0	0	0	0.05	0	0.7	0.25
**12o**	0	0	0	0.02	0.24	0.03	0.7
**12r**	0	0	0	0	0.26	0.14	0.6
**13b**	0	0	0	0	0.99	0	0.01
**13l**	0	0	0	0	1	0	0
**13m**	0	0	0	0	1	0	0
**14r**	0	0	0	0	0.78	0	0.22
**44**	0	0	0.45	0	0	0.34	0.21
**45A**	0	0	0	0	0	0.25	0.75
**45B**	0	0	0	0	0	0.62	0.38
**4a**	0	0.87	0.1	0.03	0	0	0
**4m**	0	1	0	0	0	0	0
**4p**	0	0.99	0	0.01	0	0	0
**8Ad**	0	0	0	0.27	0	0.72	0.01
**8Av**	0	0	0.01	0	0	0.98	0.01
**8Bd**	0	0	0	0	0	0	1
**8Bm**	0	0	0	0	0	0	1
**8Bs**	0	0	0	0.25	0	0.35	0.4
**9d**	0	0	0	0.14	0	0.08	0.78
**9m**	0	0	0	0	0	0	1
**a46d**	0	0	0	0	0	0.53	0.47
**a46dr**	0	0	0	0	0.05	0.27	0.67
**a46v**	0	0	0	0	0	0.79	0.21
**a46vr**	0	0	0	0	0.07	0.25	0.68
**F2d**	0	0.21	0.48	0.07	0	0.24	0
**F2v**	0	0.02	0.5	0	0	0.18	0.3
**F3**	0	0.3	0	0.42	0	0.06	0.22
**F4d**	0	0.36	0.63	0.02	0	0	0
**F4s**	0	0	0.68	0	0	0.32	0
**F4v**	0	0.1	0	0.9	0	0	0
**F5d**	0	0	0	0.67	0	0.22	0.12
**F5s**	0	0	0.44	0.12	0	0.13	0.31
**F5v**	0	0	0	0.35	0	0.08	0.57
**F6**	0	0	0	0	0	0.23	0.77
**F7d**	0	0	0	0	0	0.18	0.82
**F7i**	0	0	0	0	0	0.11	0.89
**F7s**	0	0	0	0	0	0.08	0.92
**p46d**	0	0	0	0	0	1	0
**p46dr**	0	0	0	0.58	0	0.42	0
**p46v**	0	0	0	0	0	1	0
**p46vr**	0	0	0	0	0	0.67	0.33
**1**	0	0.92	0	0.08	0	0	0
**2**	0	0.98	0	0.02	0	0	0
**3al**	0	0.81	0	0.17	0	0.01	0.02
**3am**	0	1	0	0	0	0	0
**3bl**	0	0.93	0	0.06	0	0	0
**3bm**	0	1	0	0	0	0	0
**AIP**	0	0.05	0.95	0	0	0	0
**DP**	0.42	0	0.55	0	0	0.03	0

**LIPd**	0	0	0.82	0	0	0.18	0
**LIPv**	0	0	1	0	0	0	0
**LOP**	0	0	1	0	0	0	0
**MIPd**	0	0.47	0.53	0	0	0	0
**MIPv**	0	0.06	0.94	0	0	0	0
**Opt**	0	0	0.29	0	0	0.71	0
**PEc**	0	0.31	0.69	0	0	0	0
**PEci**	0	1	0	0	0	0	0
**PEipe**	0	0.79	0.21	0	0	0	0
**PEipi**	0	0.08	0.92	0	0	0	0
**PEl**	0	0.92	0.08	0	0	0	0
**PEla**	0	1	0	0	0	0	0
**PF**	0	0.23	0.43	0.34	0	0	0
**PFG**	0	0	1	0	0	0	0
**PFop**	0	0.36	0.06	0.58	0	0	0
**PG**	0	0	0.75	0	0	0.25	0
**PGm**	0	0	0.3	0.5	0	0.02	0.18
**PGop**	0	0	0.65	0.31	0	0.04	0
**PIP**	0.02	0	0.98	0	0	0	0
**PPt**	0	0	0.02	0	0	0.98	0
**TSA**	0	1	0	0	0	0	0
**V6Adl**	0	0	1	0	0	0	0
**V6Adm**	0	0	0.31	0	0	0.4	0.29
**V6Avl**	0	0	0.95	0	0	0.05	0
**V6Avm**	0.01	0	0.01	0	0	0.53	0.45
**V6l**	0.57	0	0.43	0	0	0	0
**V6m**	0.72	0	0.04	0	0	0.23	0
**VIP**	0	0.02	0.98	0	0	0	0
**V1**	1	0	0	0	0	0	0
**V2d**	0.92	0	0	0	0	0	0.08
**V2v**	0.99	0	0	0	0	0	0
**V3A**	0.95	0	0.05	0	0	0	0
**V3d**	0.92	0	0.08	0	0	0	0
**V3v**	1	0	0	0	0	0	0
**V4dl**	0.96	0	0.04	0	0	0	0
**V4dm**	0.24	0	0.76	0	0	0	0
**V4v**	0.92	0	0	0	0	0	0.06
**23c**	0	0.96	0	0.04	0	0	0
**23d**	0	0.33	0	0.55	0	0.06	0.06
**24ab**	0	0	0	0	0	0	1
**24c**	0	0	0	0	0	0	1
**24d**	0	0.26	0	0.68	0	0.06	0
**25**	0	0	0	0	0.74	0	0.26
**29/30**	0	0	0	0.02	0	0	0.5
**31**	0	0.63	0.04	0.33	0	0	0
**a24ab**	0	0	0	0.29	0	0.17	0.54
**a24c**	0	0	0	0.06	0	0.38	0.56
**d23ab**	0	0	0	0.51	0	0.02	0.48
**p24ab**	0	0.01	0	0.94	0	0.06	0
**p32**	0	0	0	0	0	0	1
**s32**	0	0	0	0	0.08	0	0.92
**v23ab**	0	0	0	0	0	0	0.96
**MT**	0.07	0.09	0.21	0.11	0	0.09	0.44

Supplementary Table 4: Incubation protocols.

Transmitter	Receptor	Ligand<br>(nM)	K_D<br>(nM)	Displacer	Incubation buffer	Pre-incubation	Main incubation	Final rinsing
Glutamate	AMPA	[^{3}H] AMPA<br>(10.0)	10	Quisqualate<br>(10 µM)	50 mM Tris-acetate (pH 7.2)<br>+ 100 mM KSCN*	3x10 min,<br>4°C	45 min,<br>4°C	1) 4x4 sec<br>2) Acetone/glutaraldehyde (100 ml + 2,5 ml), 2x2 sec. [illegible]
	NMDA	[^{3}H] MK801<br>(3.3)	5	(+)MK-801<br>(100 µM)	50 mM Tris-acetate (pH 7.2)<br>+ 50 µM glutmate + 30 µM glycine* + 50 µM spermidine*	15 min,<br>4°C	60 min,<br>22°C	1) 2x5 min, 4°C<br>2) Distilled water, 1x22°C
	Kainate	[^{3}H] Kainate<br>(9.4)	12	SYM 2081<br>(100 µM)	50 mM Tris-acetate (pH 7.2)<br>+ 10 mM Ca^{2+}-acetate*	3x10 min, 4°C	45 min,<br>4°C	1) 3x4 sec<br>2) Acetone/glutaraldehyde (100 ml + 2,5 ml), 2x2 sec. [illegible]
GABA	$GABA_A$	[^{3}H] Muscimol<br>(7.7)	6	GABA<br>(10 µM)	50 mM Tris-citrate (pH 7.0)	3x5 min,<br>4°C	40 min,<br>4°C	1) 3x3 sec, 4°C<br>2) Distilled water, 1x22°C
	$GABA_B$	[^{3}H] CGP 54626<br>(2.0)	1.48	CGP 55845<br>(100 µM)	50 mM Tris-HCl (pH 7.2)<br>+ 2.5 mM $CaCl_2$	3x5 min,<br>4°C	60 min,<br>4°C	1) 3x2 sec, 4°C<br>2) Distilled water, 1x22°C
	BZ	[^{3}H] Flumazenil<br>(1.0)	2	Clonazepam<br>(2 µM)	170 mM Tris-HCl (pH 7.4)	15 min,<br>4°C	60 min,<br>4°C	1) 2x1 min, 4°C<br>2) Distilled water, 1x22°C
Acetylcholine	M_1	[^{3}H] Pirenzepine<br>(1.0)	3	Pirenzepine<br>(2 µM)	Modified Krebs buffer (pH 7.4)	15 min,<br>4°C	60 min,<br>4°C	1) 2x1 min, 4°C<br>2) Distilled water, 1x22°C
	M_2	[^{3}H] Oxotremorine-M<br>(1.7)	0.8	Carbacol<br>(10 µM)	20 mM HEPES-Tris (pH 7.5)<br>+ 10 mM $MgCl_2$ + 300 nM Pirenzepine	20 min,<br>22°C	60 min,<br>22°C	1) 2x2 min, 4°C<br>2) Distilled water, 1x22°C
	M_3	[^{3}H] 4-DAMP<br>(1.0)	0.2	Atropine sulfate<br>(10 µM)	50 mM Tris-HCl (pH 7.4)<br>+ 0.1 mM PSMF + 1mM EDTA	15 min,<br>22° C	45 min,<br>22° C	1) 2x5 min, 4° C<br>2) distilled water, 1x22°C
Noradrenaline	α_1	[^{3}H]-Prazosin<br>(0.2)	0.2	Phentolamine Mesylate<br>(10 µM)	50 mM Na/K-phosphate buffer<br>(pH 7.4)	15 min,<br>22°C	60 min,<br>22°C	1) 2x5 min, 4°C<br>2) Distilled water, 1x22°C
	α_2	[^{3}H] UK 14,304<br>(0,64)	1.4	Phentolamine Mesylate<br>(10 µM)	50 mM Tris-HCl (pH 7.7)<br>+ 100 µM $MnCl_2$	15 min,<br>22°C	90 min,<br>22°C	1) 5 min, 4°C<br>2) Distilled water, 1x22°C
Serotonin	$5\text{-}HT_{1A}$	[^{3}H] 8-OH-DPAT<br>(1.0)	2	5-Hydroxy-tryptamine<br>(1 µM)	170 mM Tris-HCl (pH 7.4)<br>+ 4 mM $CaCl_2$* + 0.01% ascorbate*	30 min,<br>22°C	60 min,<br>22°C	1) 5 min, 4°C<br>2) Distilled water, 3x22°C
	$5\text{-}HT_{2A}$	[^{3}H] Ketanserin<br>(1.14)	0.5	Mianserin<br>(10 µM)	170 mM Tris-HCl (pH 7.7)	30 min,<br>22°C	120 min,<br>22°C	1) 2x10 min, 4°C<br>2) Distilled water, 3x22°C

Dopamine	D_1	$[^3H]$ SCH 23390 (1.67)	0.14	SKF 83566 (1 µM)	50 mM Tris-HCl (pH 7.4) + 120 mM NaCl + 5 mM KCl + 2 mM $CaCl_2$ + 1 mM $MgCl_2$	20 min, 22°C	90 min, 22°C	1) 2x20 min, 4°C<br>2) Distilled water, 1x22°C

* Compound only included in buffer solution for the in the main incubation. Note, that although 4-DAMP is classified as a selective M_3 receptor antagonist, it shows a comparable affinity to the M_4 receptor and a slightly lower affinity for the M_1 and M_5 subtypes [1,2]. On a similar note, the quinazoline derivative ketanserin is considered a *preferential* 5-HT_{2A} antagonist, because it also has a weak antagonistic effect on the noradrenergic α_1 receptor and the histamine H_1 receptor [3]. Finally, SCH-23390 is classified as a highly selective D_1 receptor antagonist, although it also binds to the D_5 receptor, albeit with a considerably lower affinity [4].

References:

1. Caulfield, M. P. & Birdsall, N. J. International Union of Pharmacology. XVII. Classification of muscarinic acetylcholine receptors. *Pharmacological reviews* **50**, 279–290 (1998).
2. Myslivecek, J. Multitargeting nature of muscarinic orthosteric agonists and antagonists. *Frontiers in Physiology* **13**, (2022).
3. Pietzsch, H.-J. *et al.* Synthesis and autoradiographic evaluation of a novel high-affinity Tc-99m ligand for the 5-HT2A receptor. *Nuclear medicine and biology* **26**, 865–875 (1999).
4. Demchyshyn, L. L., McConkey, F. & Niznik, H. B. Dopamine D5 receptor agonist high affinity and constitutive activity profile conferred by carboxyl-terminal tail sequence. *Journal of Biological Chemistry* **275**, 23446–23455 (2000).
