# Supplementary material for: Gradients of neurotransmitter receptor expression in the macaque cortex
Source: Nat Neurosci. 2023 Jun 19;26(7):1281–94. doi: 10.1038/s41593-023-01351-2 (PMC10322721; doi:10.1038/s41593-023-01351-2)
Supplement: Supplementary file 2 — Reporting Summary [file 41593_2023_1351_MOESM2_ESM.pdf]

nature research

Corresponding author(s): Prof. Nicola Palomero-Gallagher

Last updated by author(s): Apr 26, 2023

Reporting Summary

Nature Research wishes to improve the reproducibility of the work that we publish. This form provides structure for consistency and transparency in reporting. For further information on Nature Research policies, see our Editorial Policies and the Editorial Policy Checklist.

Statistics

For all statistical analyses, confirm that the following items are present in the figure legend, table legend, main text, or Methods section.

n/a	Confirmed	
☐	☒	The exact sample size (*n*) for each experimental group/condition, given as a discrete number and unit of measurement
☐	☒	A statement on whether measurements were taken from distinct samples or whether the same sample was measured repeatedly
☐	☒	The statistical test(s) used AND whether they are one- or two-sided<br>*Only common tests should be described solely by name; describe more complex techniques in the Methods section.*
☒	☐	A description of all covariates tested
☐	☒	A description of any assumptions or corrections, such as tests of normality and adjustment for multiple comparisons
☐	☒	A full description of the statistical parameters including central tendency (e.g. means) or other basic estimates (e.g. regression coefficient) AND variation (e.g. standard deviation) or associated estimates of uncertainty (e.g. confidence intervals)
☐	☒	For null hypothesis testing, the test statistic (e.g. *F*, *t*, *r*) with confidence intervals, effect sizes, degrees of freedom and *P* value noted<br>*Give P values as exact values whenever suitable.*
☒	☐	For Bayesian analysis, information on the choice of priors and Markov chain Monte Carlo settings
☒	☐	For hierarchical and complex designs, identification of the appropriate level for tests and full reporting of outcomes
☐	☒	Estimates of effect sizes (e.g. Cohen's *d*, Pearson's *r*), indicating how they were calculated

Our web collection on statistics for biologists contains articles on many of the points above.

Software and code

Policy information about availability of computer code

Data collection	Software: Axiovision (4.8.2), Matlab (R2019b), Systat (v13), Human Connectome Project software (connectome workbench software, v1.5.0), Registration Fusion (RF) v0.18.1 (https://github.com/ThomasYeoLab/CBIG/tree/master/stable_projects/registration/Wu2017_RegistrationFusion); Advanced Normalization Tools (ANTs; version RF-ANTs); Freesurfer (v7)<br>Online Data sources: https://core-nets.org; https://balsa.wustl.edu/study/W336; https://surfer.nmr.mgh.harvard.edu/fswiki/AllenBrainAtlas; Allen Brain Map; https://search.kg.ebrains.eu/instances/e39a0407-a98a-480e-9c63-4a2225ddfbe4; human meta-analysis database (https://neuroquery.org/)
Data analysis	Code used for data integration and analysis in this study is available on GitHub (github.com/seanfw/receptorgradients, github.com/seanfw/genemapper).

For manuscripts utilizing custom algorithms or software that are central to the research but not yet described in published literature, software must be made available to editors and reviewers. We strongly encourage code deposition in a community repository (e.g. GitHub). See the Nature Research guidelines for submitting code & software for further information.

Data

Policy information about availability of data

All manuscripts must include a data availability statement. This statement should provide the following information, where applicable:

- Accession codes, unique identifiers, or web links for publicly available datasets
- A list of figures that have associated raw data
- A description of any restrictions on data availability

Data used for the analyses presented in this study is available in table format in the Source data flie associated with the article (FroudistWalsh_SourceData_Fig1.xlsx). It is also available via BALSA (Study ID: P2Nql, https://balsa.wustl.edu/study/P2Nql) and the EBRAINS platform (https://

doi.org/10.25493/5HK3-S8M)
Online Data sources: https://core-nets.org; https://balsa.wustl.edu/study/W336; https://surfer.nmr.mgh.harvard.edu/fswiki/AllenBrainAtlas; Allen Brain Map; https://search.kg.ebrains.eu/instances/e39a0407-a98a-480e-9c63-4a2225ddfbe4; human meta-analysis database (https://neuroquery.org/)

Field-specific reporting

Please select the one below that is the best fit for your research. If you are not sure, read the appropriate sections before making your selection.

☒ Life sciences ☐ Behavioural & social sciences ☐ Ecological, evolutionary & environmental sciences

For a reference copy of the document with all sections, see nature.com/documents/nr-reporting-summary-flat.pdf

Life sciences study design

All studies must disclose on these points even when the disclosure is negative.

Sample size	n = 3 for receptor data from Macaca fascicularis specimens. n = 6 for data from rat specimens. n = 5 for data from human specimens. Other data was previously described elsewhere (cited in manuscript). No statistical methods were used to pre-determine sample sizes but our sample sizes are similar to those reported in previous publications (cited in the manuscript)
Data exclusions	None.
Replication	None. A replication would require the sacrifice of further monkeys and rats, and this would not be ethically justifiable
Randomization	N/A. We analyzed data from a single experimental group. Thus, randomization was not necessary.
Blinding	N/A. We analyzed data from a single experimental group. Thus, randomization was not necessary.

Reporting for specific materials, systems and methods

We require information from authors about some types of materials, experimental systems and methods used in many studies. Here, indicate whether each material, system or method listed is relevant to your study. If you are not sure if a list item applies to your research, read the appropriate section before selecting a response.

Materials & experimental systems

n/a	Involved in the study	
☒	☐	Antibodies
☒	☐	Eukaryotic cell lines
☒	☐	Palaeontology and archaeology
☐	☒	Animals and other organisms
☐	☒	Human research participants
☒	☐	Clinical data
☒	☐	Dual use research of concern

Methods

n/a	Involved in the study	
☒	☐	ChIP-seq
☒	☐	Flow cytometry
☐	☒	MRI-based neuroimaging

Animals and other organisms

Policy information about studies involving animals; ARRIVE guidelines recommended for reporting animal research

Laboratory animals	Adult (8, 7 and 7 years old) male Macaca fascicularis specimens. Adult (exact age not available) male Lewis rats.
Wild animals	The study did not involve wild animals.
Field-collected samples	The study did not involve samples collected in the field.
Ethics oversight	The procedures used in this study had the approval of the Institutional Animal Care and Use Committee and were carried out in accordance with the European Council Directive of 2010. Covance Preclinical Services GmbH

Note that full information on the approval of the study protocol must also be provided in the manuscript.

Human research participants

Policy information about studies involving human research participants

Population characteristics	We examined a total of 5 post-mortem human brains from subjects (76±3 years of age; 3 males) without a history of neurological or psychiatric diseases.

Recruitment	Brain samples were obtained through the body donor program of the Department of Anatomy, University of Düsseldorf, Germany.
Ethics oversight	The use of these samples was approved by the Ethics board of the Medical Faculty of the University of Düsseldorf, Germany.

Note that full information on the approval of the study protocol must also be provided in the manuscript.

Magnetic resonance imaging

Experimental design

Design type	We re-used previously published and publicly available structural and resting-state functional MRI datasets.
Design specifications	Full description of the MRI data is available in the papers by Xu et al., NeuroImage, 2020 and Glasser and Van Essen, J. Neurosci, 2011 as cited in the manuscript .
Behavioral performance measures	N/A.

Acquisition

Imaging type(s)	structural (T1w/T2w) and resting-state fMRI
Field strength	Full description of the MRI data is available in the papers by Xu et al., NeuroImage, 2020 and Glasser and Van Essen, J. Neurosci, 2011 as cited in the manuscript .
Sequence & imaging parameters	Full description of the MRI data is available in the papers by Xu et al., NeuroImage, 2020 and Glasser and Van Essen, J. Neurosci, 2011 as cited in the manuscript .
Area of acquisition	whole brain.

Diffusion MRI ☐ Used ☒ Not used

Preprocessing

Preprocessing software	We re-used fully pre-processed data. Preprocessing is described completely in Xu et al., NeuroImage, 2020, and Glasser and Van Essen, J. Neurosci, 2011, as cited in manuscript.
Normalization	We re-used fully pre-processed data. Preprocessing is described completely in Xu et al., NeuroImage, 2020, and Glasser and Van Essen, J. Neurosci, 2011, as cited in manuscript.
Normalization template	We re-used fully pre-processed data. Preprocessing is described completely in Xu et al., NeuroImage, 2020, and Glasser and Van Essen, J. Neurosci, 2011 as cited in manuscript.
Noise and artifact removal	We re-used fully pre-processed data. Preprocessing is described completely in Xu et al., NeuroImage, 2020, and Glasser and Van Essen, J. Neurosci, 2011 as cited in manuscript.
Volume censoring	We re-used fully pre-processed data. Preprocessing is described completely in Xu et al., NeuroImage, 2020, and Glasser and Van Essen, J. Neurosci, 2011, as cited in manuscript.

Statistical modeling & inference

Model type and settings	We used output from the study of Xu et al., NeuroImage, 2020. They used diffusion map embedding to identify gradients of functional connectivity.
Effect(s) tested	N/A

Specify type of analysis: ☒ Whole brain ☐ ROI-based ☐ Both

Statistic type for inference (See Eklund et al. 2016)	N/A
Correction	N/A

Models & analysis

n/a	Involved in the study
☐	☒ Functional and/or effective connectivity
☒	☐ Graph analysis
☒	☐ Multivariate modeling or predictive analysis

Functional and/or effective connectivity

Thresholded functional connectivity matrices (based on Pearson correlations) were used to create joint-similarity matrices. See Methods and Xu et al., NeuroImage, 2020 for details.
